# Supplementary material for: Accounting for forest condition in Europe based on an international statistical standard
Source: Nat Commun. 2023 Jun 22;14:3723. doi: 10.1038/s41467-023-39434-0 (PMC10287664; doi:10.1038/s41467-023-39434-0)
Supplement: Supplementary file 1 — Supplementary information [file 41467_2023_39434_MOESM1_ESM.pdf]

Supplementary information

Accounting for forest condition in Europe based on an international statistical standard

Joachim Maes[1,2], Adrián G. Bruzón[3], José I. Barredo[2], Sara Vallecillo[2], Peter Vogt[2], Inés Marí Rivero[2], Fernando Santos-Martín[3]

[1]European Commission, Directorate-General for Regional and Urban Policy, Brussels, Belgium

[2]European Commission, Joint Research Centre, Ispra, Italy

[3]Department of Chemical and Environmental Technology, ESCET, Rey Juan Carlos University, Madrid, Spain

Supplementary Table 1. Forest ecosystem extent account with the opening and closing extent per region and per forest class.

Region	Forest class	Opening extent (2000, km^2)	Additions (km^2)	Reductions (km^2)	Closing extent (2018, km^2)
Alpine	Broad-leaved forest	68,571.9	589.5		69,160.4
	Coniferous forest	69,955.2	341.4		70,296.6
	Mixed forest	46,171.2			45,516.2
	Transitional woodland and shrub	19,867.5		233.2	19,634.3
Alpine (Scandinavia)	Broad-leaved forest	43,003.5	1,468.0		44,471.5
	Coniferous forest	27,827.2	1,085.0		28,912.1
	Mixed forest	6,017.7		141.0	5,876.7
	Transitional woodland and shrub	8,716.8		6,496.7	2,220.2
Arctic	Broad-leaved forest	266.8	209.5		476.2
	Coniferous forest	16.7	27.5		44.3
	Mixed forest	68.2		15.7	52.4
	Transitional woodland and shrub	223.5	133.0		356.5
Atlantic	Broad-leaved forest	67,879.1	5,959.4		73,838.5
	Coniferous forest	51,163.2	177.3		51,340.5
	Mixed forest	17,168.2		1,808.1	15,360.1
	Transitional woodland and shrub	17,870.2		1,180.9	16,689.2
Black sea	Broad-leaved forest	19,860.9	164.8		20,025.7
	Coniferous forest	13,086.6	467.2		13,553.9
	Mixed forest	14,365.7		1,642.8	12,723.0
	Transitional woodland and shrub	10,542.7		661.8	9,880.9

Boreal	Broad-leaved forest	25,033.4		2,191.4	22,842.0
	Coniferous forest	357,485.1	41,590.7		399,075.8
	Mixed forest	129,817.3		14,217.9	115,599.4
	Transitional woodland and shrub	99,233.9		20,675.3	78,558.6
Continental	Broad-leaved forest	191,751.1	12,815.1		204,566.2
	Coniferous forest	148,307.0	952.1		149,259.1
	Mixed forest	76,244.7		6,195.5	70,049.1
	Transitional woodland and shrub	30,059.5	3,036.7		33,096.2
Macaronesian	Broad-leaved forest	482.8	75.5		558.3
	Coniferous forest	922.8	25.8		948.6
	Mixed forest	131.3	21.1		152.4
	Transitional woodland and shrub	145.0	118.4		263.4
Mediterranean	Broad-leaved forest	115,667.7	3,494.0		119,161.7
	Coniferous forest	89,085.7	7,054.8		96,140.4
	Mixed forest	41,802.3		1,747.4	40,054.9
	Transitional woodland and shrub	115,385.3		12,376.4	103,008.9
Pannonian	Broad-leaved forest	19,051.8	343.1		19,394.9
	Coniferous forest	1,340.8		155.6	1,185.2
	Mixed forest	1,848.7	3.2		1,851.9
	Transitional woodland and shrub	3,305.0	1,764.1	0.0	5,069.1
Steppic	Broad-leaved forest	2,589.0	87.2		2,676.2
	Coniferous forest	2.0		0.5	1.5
	Mixed forest	7.4	1.1		8.5
	Transitional woodland and shrub	261.5		2.1	259.4
Total per class	Broad-leaved forest	554,157.0	23,014.6		57,7171.6
	Coniferous forest	759,192.4	51,565.7		81,0758.1
	Mixed forest	333,642.7		26,398.1	30,7244.6
	Transitional Woodland and Shrub	305,610.8		36,574.1	26,9036.7
Total forest area		1,952,602.9	11,608.1		1,964,211.0

Supplementary Table 2. Results of a two-tailed Mann-Whitney U test to compare the average forest condition between two years by forest type. The test is based on a random selection of 1,000 grid cells per forest type from the 2000 forest condition map and a random selection of 1,000 grid cells per forest type from the 2018 forest condition map. The statistic tests the null hypothesis that there is no difference between the average forest condition per forest type in 2000 and 2012.

Forest type	Forest condition index 2000	Forest condition index 2018	Mann-Whitney U statistic (N = 1000)	Significance level P
Alpine (Scandinavia)-Broad-leaved forest	0.550	0.577	419,815	2.66×10^{-10}
Alpine (Scandinavia)-Coniferous forest	0.613	0.650	384,676	2.12×10^{-19}
Alpine (Scandinavia)-Mixed forest	0.590	0.651	317,328	9.86×10^{-46}
Alpine (Scandinavia)-Transitional woodland and shrub	0.505	0.650	272,928	1.62×10^{-69}
Alpine-Broad-leaved forest	0.689	0.691	466,723	4.98×10^{-03}
Alpine-Coniferous forest	0.682	0.702	436,942	5.22×10^{-07}
Alpine-Mixed forest	0.705	0.721	435,464	2.90×10^{-07}
Alpine-Transitional woodland and shrub	0.610	0.611	489,944	2.18×10^{-01}
Arctic-Broad-leaved forest	0.402	0.423	8,390	3.96×10^{-05}
Arctic-Coniferous forest	0.392	0.461	96	4.74×10^{-02}
Arctic-Mixed forest	0.415	0.453	791	1.10×10^{-02}
Arctic-Transitional woodland and shrub	0.346	0.380	4,254	1.62×10^{-10}
Atlantic-Broad-leaved forest	0.496	0.531	407,637	4.26×10^{-13}
Atlantic-Coniferous forest	0.578	0.601	447,879	2.72×10^{-05}
Atlantic-Mixed forest	0.554	0.582	421,580	6.28×10^{-10}
Atlantic-Transitional woodland and shrub	0.526	0.523	455,558	2.89×10^{-04}
Black Sea-Broad-leaved forest	0.712	0.729	457,599	5.13×10^{-04}
Black Sea-Coniferous forest	0.751	0.783	430,479	3.65×10^{-08}
Black Sea-Mixed forest	0.743	0.772	442,541	4.30×10^{-06}
Black Sea-Transitional woodland and shrub	0.581	0.603	433,214	1.16×10^{-07}
Boreal-Broad-leaved forest	0.620	0.649	450,424	6.17×10^{-05}
Boreal-Coniferous forest	0.649	0.667	428,457	1.51×10^{-08}
Boreal-Mixed forest	0.633	0.648	479,076	5.26×10^{-02}
Boreal-Transitional woodland and shrub	0.643	0.630	444,118	7.54×10^{-06}
Continental-Broad-leaved forest	0.605	0.610	495,211	3.55×10^{-01}
Continental-Coniferous forest	0.642	0.651	475,250	2.76×10^{-02}
Continental-Mixed forest	0.601	0.615	477,616	4.15×10^{-02}
Continental-Transitional woodland and shrub	0.545	0.561	497,185	4.14×10^{-01}
Macaronesian-Broad-leaved forest	0.604	0.539	57,170	3.60×10^{-08}
Macaronesian-Coniferous forest	0.570	0.517	236,896	5.17×10^{-04}
Macaronesian-Mixed forest	0.574	0.527	3,730	1.80×10^{-05}
Macaronesian-Transitional woodland and shrub	0.626	0.489	4,617	4.40×10^{-08}

Mediterranean-Broad-leaved forest	0.541	0.548	469,128	8.41×10^{-03}
Mediterranean-Coniferous forest	0.535	0.539	465,287	3.59×10^{-03}
Mediterranean-Mixed forest	0.540	0.543	440,747	2.23×10^{-06}
Mediterranean-Transitional woodland and shrub	0.462	0.463	492,517	2.81×10^{-01}
Pannonian-Broad-leaved forest	0.596	0.611	472,566	1.68×10^{-02}
Pannonian-Coniferous forest	0.639	0.644	482,044	8.22×10^{-02}
Pannonian-Mixed forest	0.576	0.598	461,956	1.61×10^{-03}
Pannonian-Transitional woodland and shrub	0.485	0.528	418,482	1.37×10^{-10}
Steppic-Broad-leaved forest	0.578	0.606	461,785	1.54×10^{-03}
Steppic-Coniferous forest	0.473	0.618	1	2.70×10^{-01}
Steppic-Mixed forest	0.308	0.366	11	1.65×10^{-01}
Steppic-Transitional woodland and shrub	0.405	0.469	7,782	1.58×10^{-05}

Supplementary Table 3. European average values (standard deviations) for forest condition variables, reference levels and indicators across 44 forest ecosystem types. The indicator values are rescaled between 0 and 1 and can therefore be compared with other indicators. A table per forest type is available as an open access dataset.

**Variable descriptor**	**Variable descriptor unit**	**Variable values (observed)**		**Reference levels**		**Indicator values (rescaled)**	
		**Year 2000**	**Year 2018**	**Lower level**	**Upper level**	**Year 2000**	**Year 2018**
Water content - Normalized difference water index (NDWI)	Index between -1 and 1	0.004 (0.044)	0.008 (0.046)	-0.28 (0.11)	0.17 (0.10)	0.63 (0.13)	0.64 (0.12)
Soil organic carbon	Index between 0 and 1	0.09 (0.08)	0.15 (0.14)	0	0.63 (0.40)	0.15 (0.12)	0.24 (0.14)
Species richness of threatened forest birds	Number	3.56 (1.89)	3.76 (2.04)	0.29 (0.55)	6.85 (2.20)	0.52 (0.19)	0.55 (0.24)
Tree cover density	%	49.60 (18.08)	52.93 (16.20)	0	96.20 (5.03)	0.52 (0.18)	0.55 (0.16)
Forest productivity - Normalized difference vegetation index (NDVI)	Index between -1 and 1	0.53 (0.10)	0.57 (0.10)	-0.02 (0.12)	0.72 (0.10)	0.75 (0.11)	0.80 (0.08)
Forest connectivity	%	63.58 (14.71)	64.01 (14.61)	2.50 (2.33)	99.66 (1.43)	0.63 (0.16)	0.63 (0.15)
Landscape naturalness	%	77.97 (13.41)	78.44 (12.41)	7.50 (6.86)	100	0.76 (0.15)	0.77 (0.13)

Supplementary Table 4. Comparison of different kriging methods with an independent data set based on Pearson correlation test (N = 23 130 samples).

Lucas 2015	Spherical	Circular	Exponential	Gaussian	Linear	Independent dataset
**Spherical**	1	1	0.9999	0.9987	0.9999	0.5595
**Circular**	1	1	0.9999	0.9990	1	0.5597
**Exponential**	0.9999	0.9999	1	0.9982	0.9998	0.5591
**Gaussian**	0.9987	0.9990	0.9982	1	0.9992	0.5604
**Linear**	0.9999	1	0.9998	0.9992	1	0.5599
**Independent dataset**	0.5595	0.5597	0.5591	0.5604	0.5599	1

Supplementary Table 5. Data sources used to model species richness of threatened forest birds.

**Variables (unit)**	**Data source**	**Spatial resolution**
Species richness of threatened bird species (number)	Distribution maps Article 12 data (2008-2012) from https://sdi.eea.europa.eu/catalogue/srv/eng/catalog.search#/metadata/7c2dd14f-60b6-4009-aca8-5d20300479a9	10×10 km
Longitude and latitude (decimal degrees)	https://www.eea.europa.eu/data-and-maps/data/eea-reference-grids-2	10×10 km
Altitude (m)	https://land.copernicus.eu/pan-european/satellite-derived-products/eu-dem/eu-dem-v1-0-and-derived-products/eu-dem-v1.0?tab=download	100×100 m
Share of artificial land (%)	https://land.copernicus.eu/pan-european/corine-land-cover	100×100 m
Share of cropland (%)	https://land.copernicus.eu/pan-european/corine-land-cover	100×100 m
Share of forest (%)	https://land.copernicus.eu/pan-european/corine-land-cover	100×100 m
Share of shrubland (%)	https://land.copernicus.eu/pan-european/corine-land-cover	100×100 m
Share of fresh water (%)	https://land.copernicus.eu/pan-european/corine-land-cover	100×100 m
Share of sparsely vegetated areas (%)	https://land.copernicus.eu/pan-european/corine-land-cover	100×100 m
Share of grassland (%)	https://land.copernicus.eu/pan-european/corine-land-cover	100×100 m
Land cover diversity (Shannon Index)	https://land.copernicus.eu/pan-european/corine-land-cover	100×100 m
Mean annual temperature (°C)	https://www.worldclim.org/data/worldclim21.html	~1×1 km
Temperature seasonality (°C)	https://www.worldclim.org/data/worldclim21.html	~1×1 km
Annual precipitation (mm)	https://www.worldclim.org/data/worldclim21.html	~1×1 km
Precipitation seasonality (mm)	https://www.worldclim.org/data/worldclim21.html	~1×1 km
Mean summer NDVI (value between -1 and 1)	https://developers.google.com/earth-engine/datasets/catalog/MODIS_MCD43A4_006_NDVI	100×100 m
Range summer NDVI (value between -1 and 1)	https://developers.google.com/earth-engine/datasets/catalog/MODIS_MCD43A4_006_NDVI	100×100 m

Supplementary Table 6. Summary statistics of the final generalized linear model to predict species richness of threatened forest birds based on nine independent predictor variables. Significance testing is based on two-tailed t-test; no adjustments for multiple comparisons.

**Predictor variables**	**Estimate**	**Standard error**	**t-value**	**P-value**
Intercept	-5.929	1.164×10^{-1}	-50.92	0
Share of forest	1.704×10^{-2}	7.460×10^{-4}	22.84	2.146×10^{-114}
Share of forests (quadratic)	-1.107×10^{-4}	7.162×10^{-6}	-15.45	1.219×10^{-53}
Mean annual temperature	6.536×10^{-2}	4.934×10^{-3}	13.25	6.149×10^{-40}
Mean annual temperature (quadratic)	-9.338×10^{-3}	3.046×10^{-4}	-30.66	4.120×10^{-203}
Temperature seasonality	1.616×10^{-2}	3.136×10^{-4}	51.54	0
Temperature seasonality (quadratic)	-9.964×10^{-6}	2.182×10^{-7}	-45.65	0
Mean summer NDVI	7.972×10^{-1}	5.739×10^{-2}	13.89	9.867×10^{-44}
Share of cropland	1.057×10^{-2}	7.308×10^{-4}	14.47	2.754×10^{-47}
Share of cropland (quadratic)	-1.496×10^{-4}	8.989×10^{-6}	-16.64	6.813×10^{-62}

Supplementary Table 7. Pairwise Pearson's correlations between the seven ecosystem condition variables (N = 5,836,150, all p-values <0.001).

	Vegetation water content - NDWI	Soil organic carbon	Species richness of threatened forest birds	Tree cover density	Forest productivity – (NDVI)	Forest connectivity	Landscape naturalness
Vegetation water content - NDWI	1.00						
Soil organic carbon	0.06	1.00					
Species richness of threatened forest birds	0.18	0.10	1.00				
Tree cover density	0.06	0.08	0.12	1.00			
Forest productivity - (NDVI)	-0.14	0.04	0.05	0.36	1.00		
Forest connectivity	0.02	0.09	0.31	0.16	0.26	1.00	
Landscape naturalness	0.14	0.14	0.20	0.04	-0.08	0.61	1.00

Supplementary Table 8. Area (ha) of primary forest and protected forest per forest ecosystem type.

**Primary forest (ha)**	**Broad-leaved forest**	**Coniferous forest**	**Mixed forest**	**Transitional woodland and shrub**	**Total**
Alpine	55,229	35,731	37,325	4,325	132,610
Arctic	36	0	0	0	36
Atlantic	6,305	8,728	515	676	16,224
Black Sea	6,767	0	0	0	6,767
Boreal	123,461	723,175	331,122	151,488	1329,246
Continental	36,770	4,394	11,024	987	53,175
Macaronesian	8,809	32	461	656	9,958
Mediterranean	6,073	1,132	2,521	821	10,547
Pannonian	652	0	2	3	657
Alpine (Scandinavia)	152,988	39,182	26,567	259,028	477,765
Steppic	159	0	0	0	159
Total	397,249	812,373	409,536	417,984	2,037,143
**Protected forest (ha)**	**Broad-leaved forest**	**Coniferous forest**	**Mixed forest**	**Transitional woodland and shrub**	**Total**
Alpine	28,342	15,377	16,932	3,069	63,721
Arctic	363	0	0	0	363
Atlantic	1,060	4,086	230	86	5,462
Black Sea	62	0	15	2	79
Boreal	120,014	570,854	301,554	122,620	1,115,042
Continental	21,553	1,387	8,197	473	31,609
Macaronesian	7,905	44,512	2,047	202	54,666
Mediterranean	399,488	159,644	90,246	123,625	773,003
Pannonian	62,607	1,246	1,623	3,949	69,426
Alpine (Scandinavia)	104,896	15,259	24,696	258,715	403,566
Steppic	10,109	0	0	101	10,210
Total	756,399	812,367	445,539	512,842	2,527,147

Supplementary Table 9. Indicator weights based on a scoring of the forest ecosystem condition variables from 1 to 7 to represent their importance per criterion.

**Conceptual criteria**	**Vegetation water content - NDWI**	**Soil organic carbon**	**Species richness of threatened forest birds**	**Tree cover density**	**Forest productivity – (NDVI)**	**Forest connectivity**	**Landscape naturalness**
Intrinsic relevance	1	3	7	6	2	5	4
Instrumental relevance	4	5	3	6	7	2	1
Directional meaning	1	3	7	5	2	4	6
Sensitivity to human influence	2	1	7	6	3	5	4
Framework conformity	3	5	7	6	4	2	1
Sum of the ranks	11	17	31	29	18	18	16
Weights	0.08	0.12	0.22	0.21	0.13	0.13	0.11

Supplementary Table 10. Expected forest class based on the descriptions of the potential natural vegetation[1].

Code	Potential natural vegetation	CORINE Land Cover forest classes expected given potential natural vegetation	Comment
**A**	**Polar deserts and subnival-nival vegetation of the high mountains**		
A.1	Arctic polar deserts		No forest expected
A.2	Subnival-nival vegetation of high mountains in the boreal and nemoral zone		No forest is expected above the tree line
**B**	**Arctic tundras and alpine vegetation**		
B.1	Arctic tundra		Forest-free vegetation types in the Arctic
B.2	Alpine vegetation (Alpine grasslands, low creeping shrub, dwarf shrub and shrub vegetation, rock and scree vegetation) in the boreal, nemoral and Mediterranean zone		Forest-free vegetation types above the tree line in the Alpine and Scandinavian Alpine regions
**C**	**Subarctic, boreal and nemoral-montane open woodlands as well as subalpine and oro-Mediterranean vegetation**		
C.1	Eastern boreal open woodlands (*Betula pubescens* subsp. *czerepanovii*, *Picea obovata*, *Pinus sylvestris*)	Broad-leaved forest<br>Coniferous forest<br>Mixed forest<br>Transitional woodland and shrub	Open woodlands with 10-30% canopy closure, mainly birch in the Boreal and in across the borders with the Arctic and Scandinavian Alpine
C.2	Western boreal and nemoral-montane birch forests (*Betula pubescens s. l.*), partly with pine forests (*Pinus sylvestris*)	Broad-leaved forest<br>Coniferous forest<br>Mixed forest<br>Transitional woodland and shrub	Forests dominated by birch (birch shrub forests, birch forests and complexes of dominant birch forests with scattered pine forests) in the Boreal and along the borders with the Atlantic and Arctic
C.3	Subalpine and oro-Mediterranean vegetation (forests, scrub and dwarf shrub communities in combination with grasslands and tall-forb communities)	Coniferous forest<br>Transitional woodland and shrub	Coniferous open woodlands and scrub with prevailing pines and larches as well as juniper in the Alpine and Mediterranean and along the borders with the Atlantic, Continental and Black Sea
**D**	**Mesophytic and hygromesophytic coniferous and mixed broadleaved-coniferous forests**		
D.1	Western boreal spruce forests (*Picea abies*, *P. obovata*, P. abies x P.	Coniferous forest<br>Mixed forest	Coniferous forests in the Boreal and along the borders with the Atlantic and Scandinavian alpine

	obovata), partly with *Pinus sylvestris*, locally with birch (*Betula pubescens s. l., B. pendula*), alder (*Alnus incana*) or mixed forests		region
D.2	Eastern boreal pine-spruce (*Picea obovata, Pinus sibirica*) and fir-spruce forests (*Picea obovata, Abies sibirica*), partly with *Betula pubescens s.l.*, and *Larix sibirica*		This natural vegetation type is situated outside (east) of the accounting area
D.3	Hemiboreal spruce (*Picea abies, P. abies x P. obovata*, *P. obovata*) and fir-spruce forests (*Picea obovata, P. abies x P. obovata, Abies sibirica*) with broad-leaved trees (*Quercus robur, Tilia cordata, Ulmus glabra, Acer platanoides* and others)	Broad-leaved forest<br>Coniferous forest<br>Mixed forest<br>Transitional woodland and shrub	Coniferous forests with broadleaved trees while subtypes of D.3 include pure coniferous and broadleaved forests. Boreal region and along the borders with the Continental and Atlantic in Norway
D.4	Montane to altimontane, partly submontane fir (*Abies alba, A. nordmanniana*) and spruce forests (*Picea abies, P. omorika, P. orientalis*) in the nemoral zone	Coniferous forest	Pure coniferous forests of firs and spruces in the Alps and along the borders with the Continental and the Mediterranean
D.5	Boreal and hemiboreal pine forests (*Pinus sylvestris*), partly with *Betula pubescens s. l., Picea obovata, P. abies*	Coniferous forest<br>Mixed forest	European pine forests in the Boreal and along the borders with the Continental, Scandinavian alpine and Atlantic (in Scotland and Norway)
D.6	Montane to altimontane (subalpine) pine forests (*Pinus peuce, P. sylvestris, P. kochiana*) in the nemoral zone	Coniferous forest	Pine forests in the Alpine and along the borders with the Continental and Mediterranean
**E**	**Atlantic dwarf shrub heaths**		
E.1	Boreo-Atlantic coastal and mountain heaths	Transitional woodland and shrub	Heathlands in the Atlantic as well as in areas of other regions close to the border of the Atlantic
E.2	Northwest European coastal heaths	Transitional woodland and shrub	Heathlands in the Atlantic as well as in areas of other regions close to the border of the Atlantic
E.3	West European coastal heaths	Transitional woodland and shrub	Heathlands in the Atlantic as well as in areas of other regions close to the border of the Atlantic

E.4	Southwest European coastal heaths	Transitional woodland and shrub	Heathlands in the Atlantic as well as in areas of other regions close to the border of the Atlantic
**F**	**Mesophytic broadleaved deciduous and mixed broadleaved/conifer forests**		
F.1	Species-poor acidophilous oak and mixed oak forests (*Quercus robur, Q. petraea, Q. pyrenaica, Pinus sylvestris, Betula pendula, B. pubescens, B. pubescens subsp. celtiberica, Castanea sativa*)	Broad-leaved forest	Oak and mixed oak forests in the Continental and Atlantic and in areas bordering these regions
F.2	Mixed oak-ash forests (*Fraxinus excelsior, Quercus robur, Ulmus glabra, Quercus petraea*)	Broad-leaved forest	Mixed oak forests in the Atlantic and in areas bordering this region
F.3	Mixed oak-hornbeam forests (*Carpinus betulus, Quercus robur, Q. petraea, Tilia cordata*)	Broad-leaved forest	Mixed oak forests in the Continental, Atlantic and Pannonian and in areas bordering these regions
F.4	Lime-pedunculate oak forests (*Quercus robur, Tilia cordata, partly Acer platanoides, A. campestre, Ulmus glabra*)	Broad-leaved forest	Oak forests in the Continental (but outside the accounting area) and the Boreal and in areas bordering these regions
F.5	Beech and mixed beech forests (*Fagus sylvatica, partly F. sylvatica subsp. moesiaca, Abies alba*)	Broad-leaved forest<br>Mixed forest	Beech forests in the Atlantic, Continental and Mediterranean and in areas bordering these regions
F.6	Oriental beech forests and hornbeam-Oriental beech forests (*Fagus sylvatica subsp. orientalis, Carpinus betulus*)	Broad-leaved forest	Beech forests in the Black Sea region and in areas bordering this region
F.7	Caucasian mixed hornbeam-oak forests (*Quercus robur, Q. petraea, Q. iberica, Q. pedunculiflora, Q. macranthera, Carpinus betulus, C. orientalis* etc.)		This natural vegetation type is situated outside (east) of the accounting area
**G**	**Thermophilous mixed deciduous broadleaved forests**		
G.1	Subcontinental thermophilous (mixed) pedunculate oak and sessile oak forests (*Quercus robur, Q. petraea, Q. dalechampii,*	Broad-leaved forest<br>Mixed forest	Mixed deciduous broadleaved forests in the Continental and Pannonian regions and in areas of the Mediterranean and the Alpine that border these two regions

	Q. polycarpa, Pinus sylvestris, Acer tataricum)		
G.2	Sub-Mediterranean-subcontinental thermophilous bitter oak and Balkan oak forests, as well as mixed forests (*Quercus cerris, Q. petraea, Q. frainetto, Q. dalechampii, Q. pedunculiflora, Q. pubescens, Q. virgiliana, Q. polycarpa, Q. hartwissiana, Carpinus*)	Broad-leaved forest	Thermophilous mixed broadleaved forests in the Continental, Pannonian, Mediterranean, Alpine and Black Sea regions
G.3	Sub-Mediterranean and meso-supra-Mediterranean downy oak forests, as well as mixed forests (*Quercus pubescens, Q. virgiliana, Q. trojana, Fraxinus ornus, Ostrya carpinifolia, Carpinus orientalis*)	Broad-leaved forest	Thermophilous deciduous broadleaved oak forests in the Continental, Pannonian, Mediterranean, Alpine, Black Sea, Steppic and Atlantic regions
G.4	Iberian supra- and meso-Mediterranean *Quercus pyrenaica, Q. faginea, Q. faginea subsp. broteroi and Q. canariensis* forests	Broad-leaved forest	Deciduous to winter-green oak forests in the Mediterranean and in the Alpine and Atlantic bordering the Mediterranean in Spain
**H**	**Hygrophilous thermophytic mixed deciduous broadleaved forests**		
H.0	Hygrophilous thermophytic mixed deciduous broadleaved forests		This natural vegetation type is situated outside (east) of the accounting area
**J**	**Mediterranean sclerophyllous forests and scrub**		
J.1	Meso- and supra-Mediterranean as well as relict sclerophyllous forests (*Quercus ilex, Q. ilex subsp. rotundifolia, Q. coccifera, Q. suber, Pistacia lentiscus*)	Broad-leaved forest<br>Coniferous forest<br>Mixed forest<br>Transitional woodland and shrub	Sclerophyllous forests and xerophytic scrub in the Mediterranean and in areas in the Atlantic and Continental that border with the Mediterranean
J.2	Thermo-Mediterranean sclerophyllous forests and xerophytic scrub (*Quercus suber, Q. ilex subsp. rotundifolia, Olea europaea, Ceratonia siliqua, Periploca angustifolia, Rhamnus lycioides*)	Broad-leaved forest<br>Coniferous forest<br>Mixed forest<br>Transitional woodland and shrub	Sclerophyllous forests and xerophytic scrub in the Mediterranean
**K**	**Xerophytic coniferous forests, coniferous woodland and scrub**		

K.1	Pine forests and pine woodlands (*Pinus sylvestris, P. nigra agg., P. heldreichii, P. halepensis, P. brutia, P. pityusa*)	Coniferous forest<br>Transitional woodland and shrub	Xerophytic coniferous forests, woodland and scrub in the Alpine, Mediterranean and Continental
K.2	Meso- and supra-Mediterranean fir forests (*Abies pinsapo, A. cephalonica*)	Coniferous forest	Xerophytic fir forests in the Mediterranean (Balkans and southern Spain)
K.3	Juniper and cypress open woodlands and scrub (*Juniperus thurifera, J. excelsa, J. foetidissima, J. polycarpos, Cupressus sempervirens*)	Coniferous forest<br>Transitional woodland and shrub	Open woodlands with arborescent juniper species occur scattered throughout wide areas of the Mediterranean region
**L**	**Forest steppes (Meadow steppes alternating with nemoral deciduous forests) and dry grasslands alternating with dry scrub**		
L.1	Subcontinental meadow steppes and steppe-like dry grasslands (*Festuca rupicola, F. valesiaca, Stipa tirsa, S. pennata, Poa angustifolia, Agrostis vinealis*), alternating with pedunculate oak forests (*Quercus robur*)	Broad-leaved forest<br>Coniferous forest<br>Transitional woodland and shrub	Mesophilic broadleaved forests occurring on sandy soils (also pine forests) in the Continental and in areas of the Steppic along the border with the Continental
L.2	Sub-Mediterranean-subcontinental herb-grass steppes, partly meadow steppes (*Festuca valesiaca, Stipa spp., Bothriochloa ischaemum, Chrysopogon gryllus*), alternating with oak forests (*Quercus pubescens, Q. robur, Q. pedunculiflora*) with *Acer tataricum*	Broad-leaved forest<br>Transitional woodland and shrub	Dry oak forests on sandy soils in the Pannonian, Continental, Steppic, Black Sea and Mediterranean
**M**	**Steppes**		
M.1	True steppes (*Stipa pennata, S. tirsa, S. dasyphylla, S. ucrainica, Festuca valesiaca, Koeleria macrantha*)		Steppes are naturally treeless grasslands occurring in the Steppic region and in border areas with Continental and Black Sea
M.2	Desert steppes (*Stipa lessingiana, S. sareptana, Festuca valesiaca, Artemisia spp.*)		This natural vegetation type is situated outside (east) of the accounting area
**N**	**Oroxerophytic vegetation (thorn-cushion communities, tomillares, mountain steppes, in part scrub)**		
N.0	Oroxerophytic vegetation	Transitional woodland and	Woodland and shrub in the

	(thorn-cushion communities, tomillares, mountain steppes, in part scrub)	shrub	Mediterranean, Black Sea and Continental
**O**	**Deserts**		
O.1	Deserts		This natural vegetation type is situated outside (east) of the accounting area
**P**	**Coastal vegetation and inland halophytic vegetation**		
P.1	Vegetation of coastal sand dunes and sea shores, often in combination with halophytic vegetation, partly with vegetation of rocky sea shores	Broad-leaved forest<br>Coniferous forest<br>Mixed forest<br>Transitional woodland and shrub	Forests along the coastal shores of the Atlantic, Mediterranean, Black Sea, Continental, Boreal, Steppic and Arctic
P.2	Halophytic vegetation		Treeless vegetation types along the coastal shores of the Atlantic, Mediterranean, Black Sea, Continental, Boreal, Steppic and Arctic
**R**	**Tall reed vegetation and tall sedge swamps, aquatic vegetation**		
R1, R2, R3, R4	Tall reed vegetation and tall sedge swamps, aquatic vegetation	No forest class assigned	Treeless vegetation types in the Boreal, Atlantic, Mediterranean, Pannonian, Steppic and Black Sea
**S**	**Mires**		
S.1	Ombrotrophic mires (bogs)	Broad-leaved forest<br>Coniferous forest<br>Mixed forest<br>Transitional woodland and shrub	Forests on raised bods in the Atlantic, Alpine, Scandinavian Alpine, Continental and Boreal
S.2	Ombro-minerotrophic mires		Treeless vegetation (under permafrost) in the Arctic, Boreal and Scandinavian Alpine
S.3	Minerotrophic mires (fens)		Treeless waters in the Arctic, Boreal, Scandinavian Alpine, Continental, Pannonian, Alpine and Atlantic
**T**	**Swamp and fen forests**		
T.1	Alder carrs and swamp forests (*Alnus glutinosa, A. barbata*)	Broad-leaved forest	Deciduous swamp and fen forests consisting solely or predominantly of black alder in the Atlantic, Continental, and Boreal
T.2	Birch carrs and swamp forests (*Betula pubescens s. l.*)	Broad-leaved forest	Deciduous carrs and swamp forests of downy birch in the Atlantic, Continental and Boreal

T.3	Vegetation complexes on degraded lowland raised bogs	Broad-leaved forest	Moist to wet downy birch forests in the Atlantic
**U**	**Vegetation of floodplains, estuaries, fresh-water polders and other moist or wet sites**		
U.1	Southern arctic alluvial scrub (*Salix phylicifolia, Alnus fruticosa*)		This natural vegetation type is situated outside (north-east) of the accounting area
U.2	Boreal alluvial forests	Broad-leaved forest<br>Coniferous forest<br>Mixed forest	Coniferous, mixed deciduous and broadleaved alluvial forests in the Boreal and Arctic, but mostly outside the accounting area
U.3	Alluvial and wet lowland forests in the nemoral zone	Broad-leaved forest	Deciduous broadleaved trees in the Atlantic, Continental, Steppic, Pannonian, Alpine, Mediterranean, and Black Sea as well as in areas in the Boreal close to border with the Continental
U.4	Mediterranean-sub-Mediterranean wet lowland and alluvial forests and scrub (*Fraxinus angustifolia s. l., F. pallisae, Platanus orientalis, Phoenix theophrasti, Nerium oleander, Tamarix spp*.)	Broad-leaved forest<br>Transitional woodland and shrub	Alluvial forests and scrub as well as groundwater-influenced lowland forests in the Mediterranean and in areas of Atlantic, Alpine, and Continental that border the Mediterranean.
U.5	Continental softwood alluvial forests (*Populus nigra, P. alba, Salix alba*) and tamarisk alluvial scrub (*Tamarix ramosissima*)	Broad-leaved forest<br>Transitional woodland and shrub	Forests along rivers in the Steppic but mostly outside the accounting area

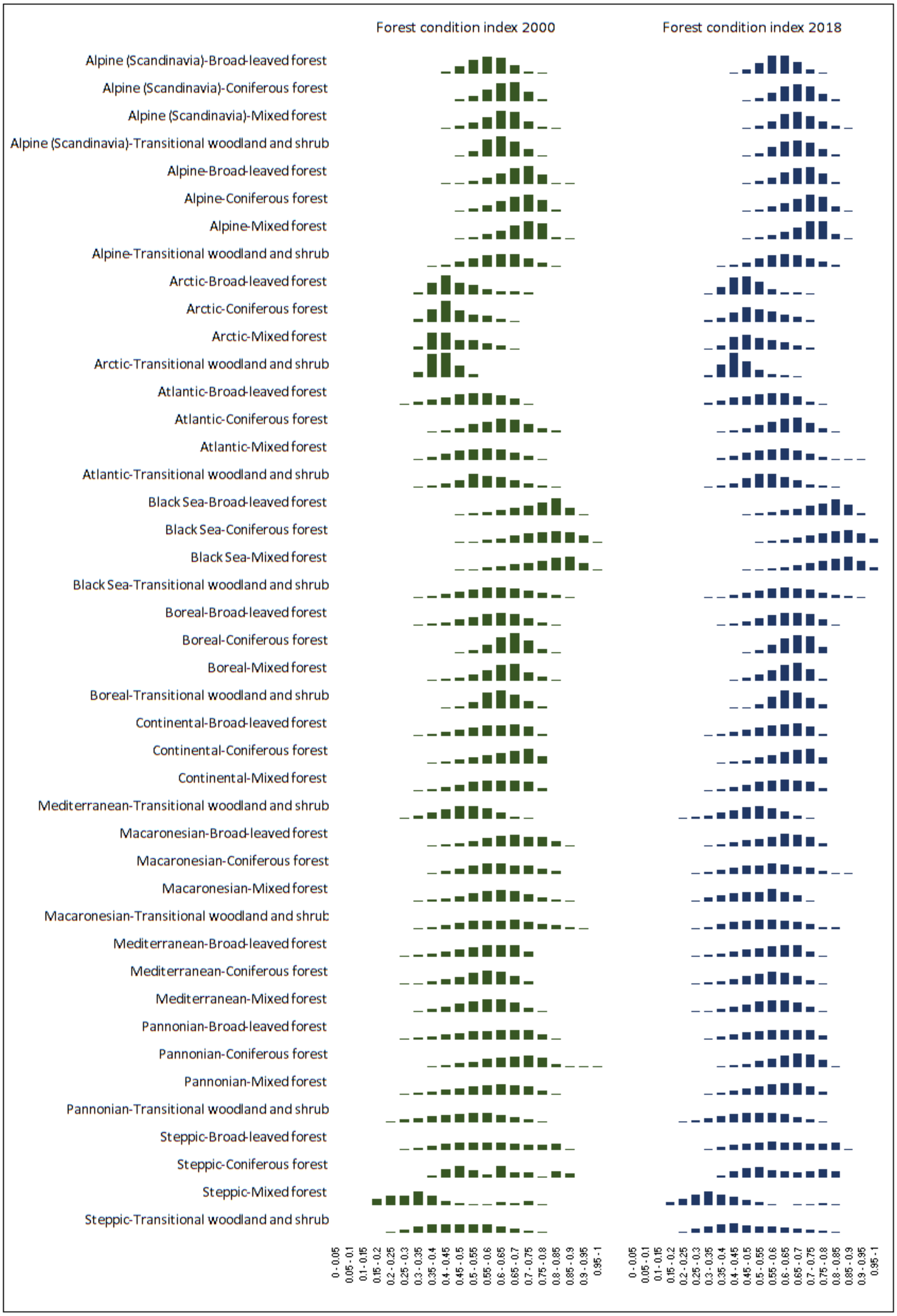

Supplementary Figure 1. Distribution of the forest condition index by forest type. The highest bar represents a relative frequency of 36%.

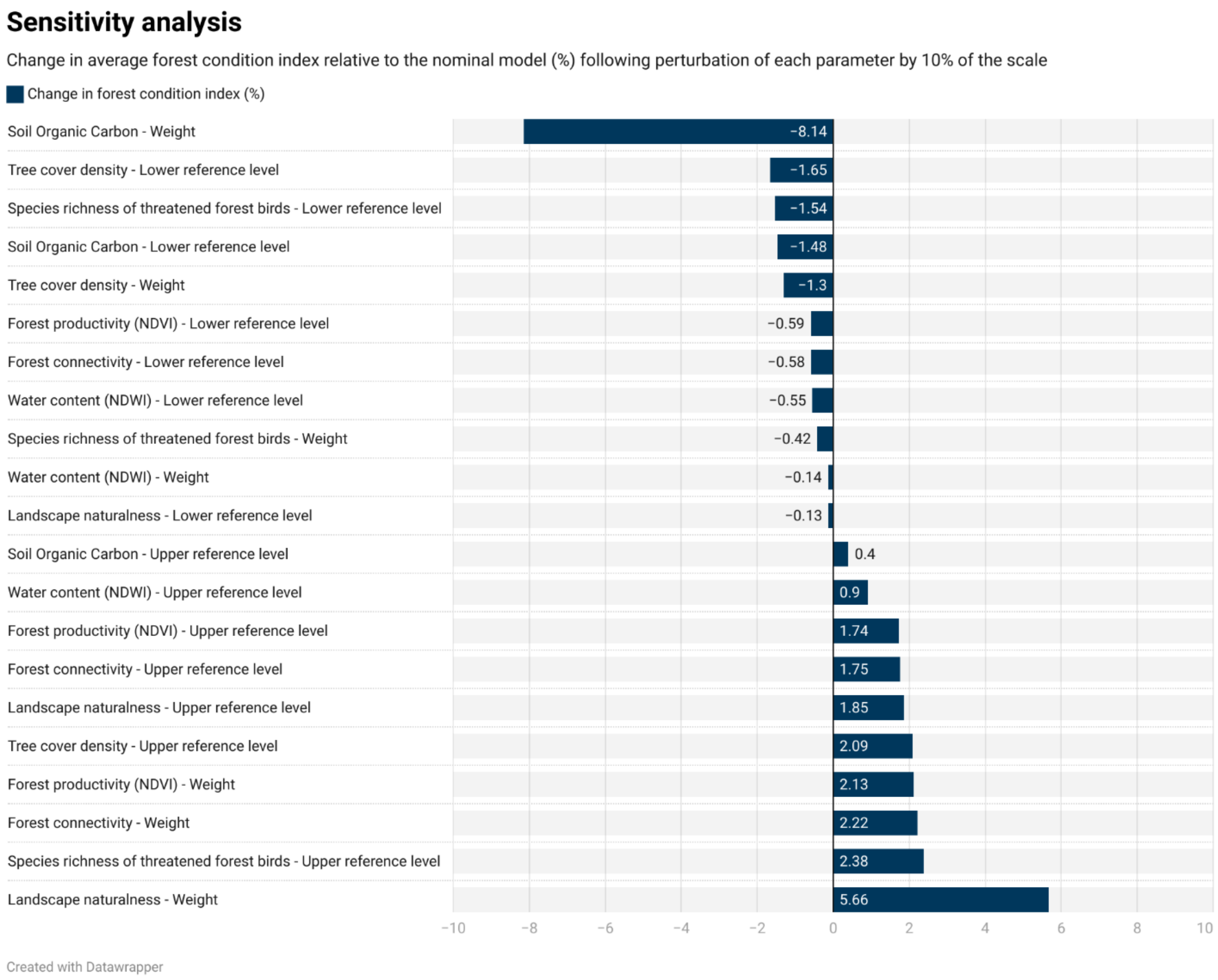

Supplementary Figure 2. Results of the sensitivity analysis. This figure shows the average deviation in the forest condition index following the perturbation of the parameters with 10% of their value. The analysis was done for every forest type. This figure presents the average deviations of the forest condition index.

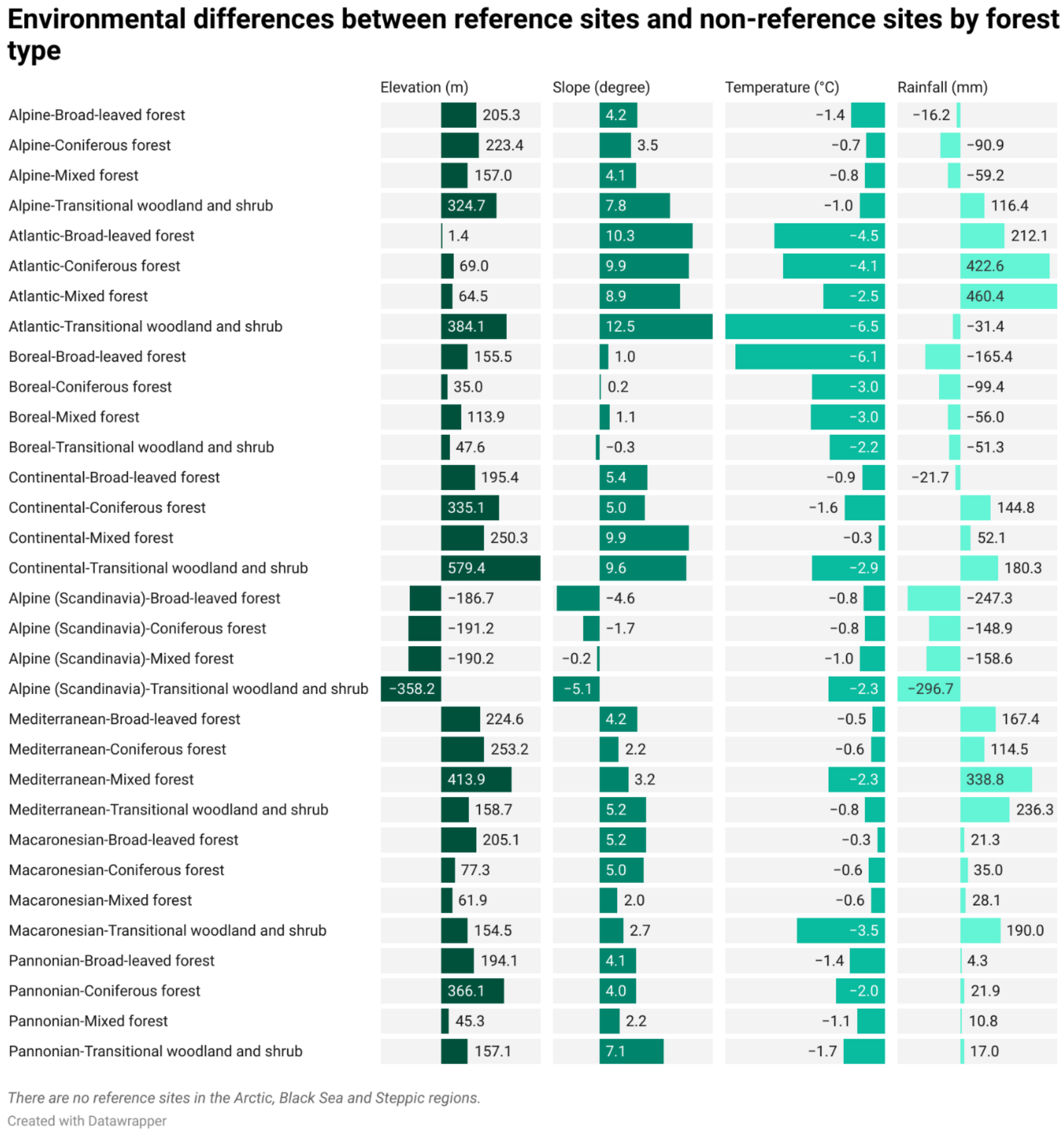

Supplementary Figure 3. Differences in elevation, slope, annual average temperature and annual precipitation between reference sites and non-reference sites by forest type. Positive values mean that reference sites have higher values than non-reference sites.

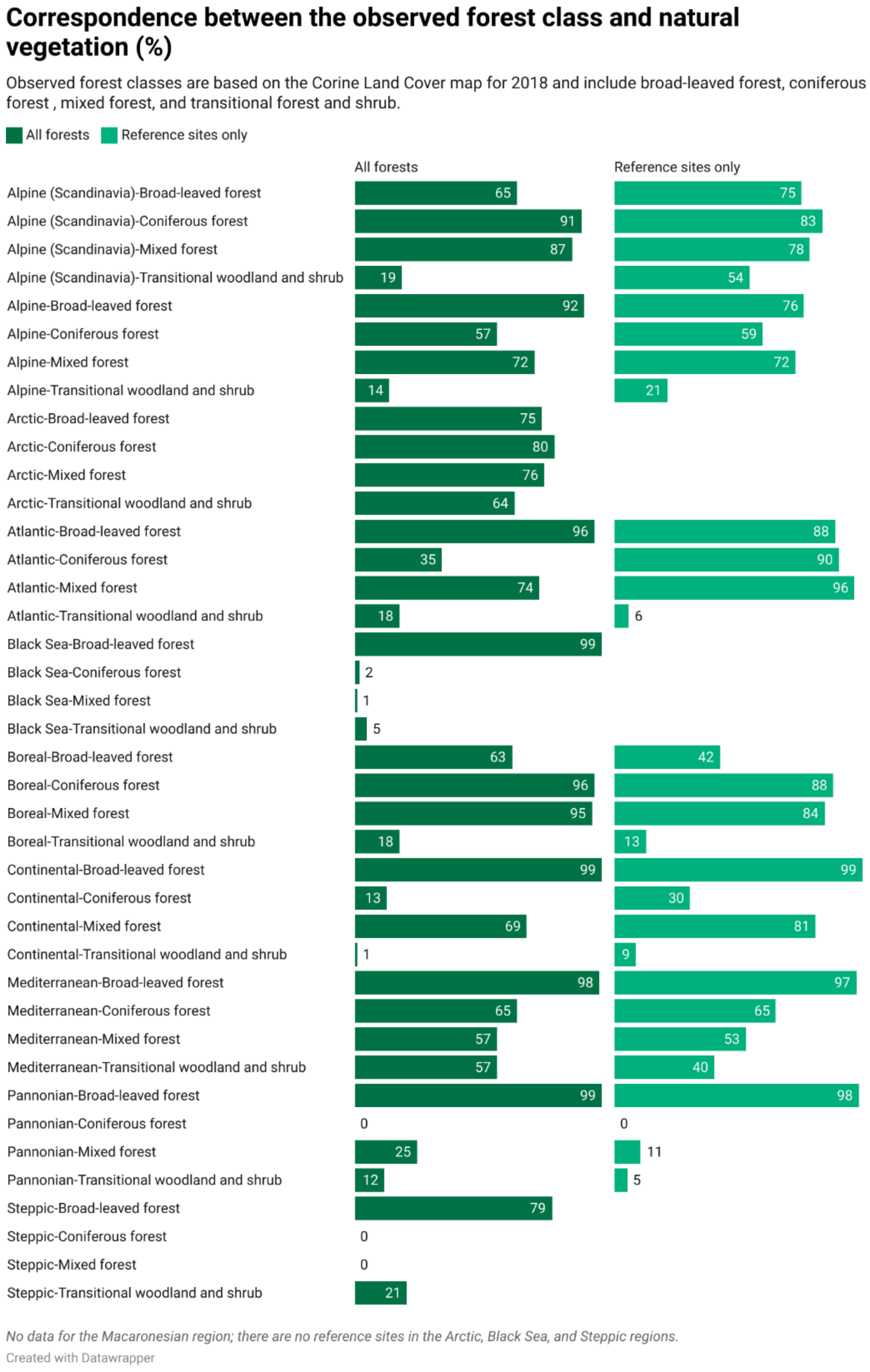

Supplementary Figure 4. Correspondence between the observed forest class and naturally occurring forest class based on potential natural vegetation for all forests and for reference forests only. Data by forest type. A correspondence of 80% means that 80% of the area covered by a specific forest type coincides with a forest class that is expected based on the natural vegetation map and that 20% of the forest area of that specific forest type occurs on a place where the map of natural vegetation does not expect that forest class.

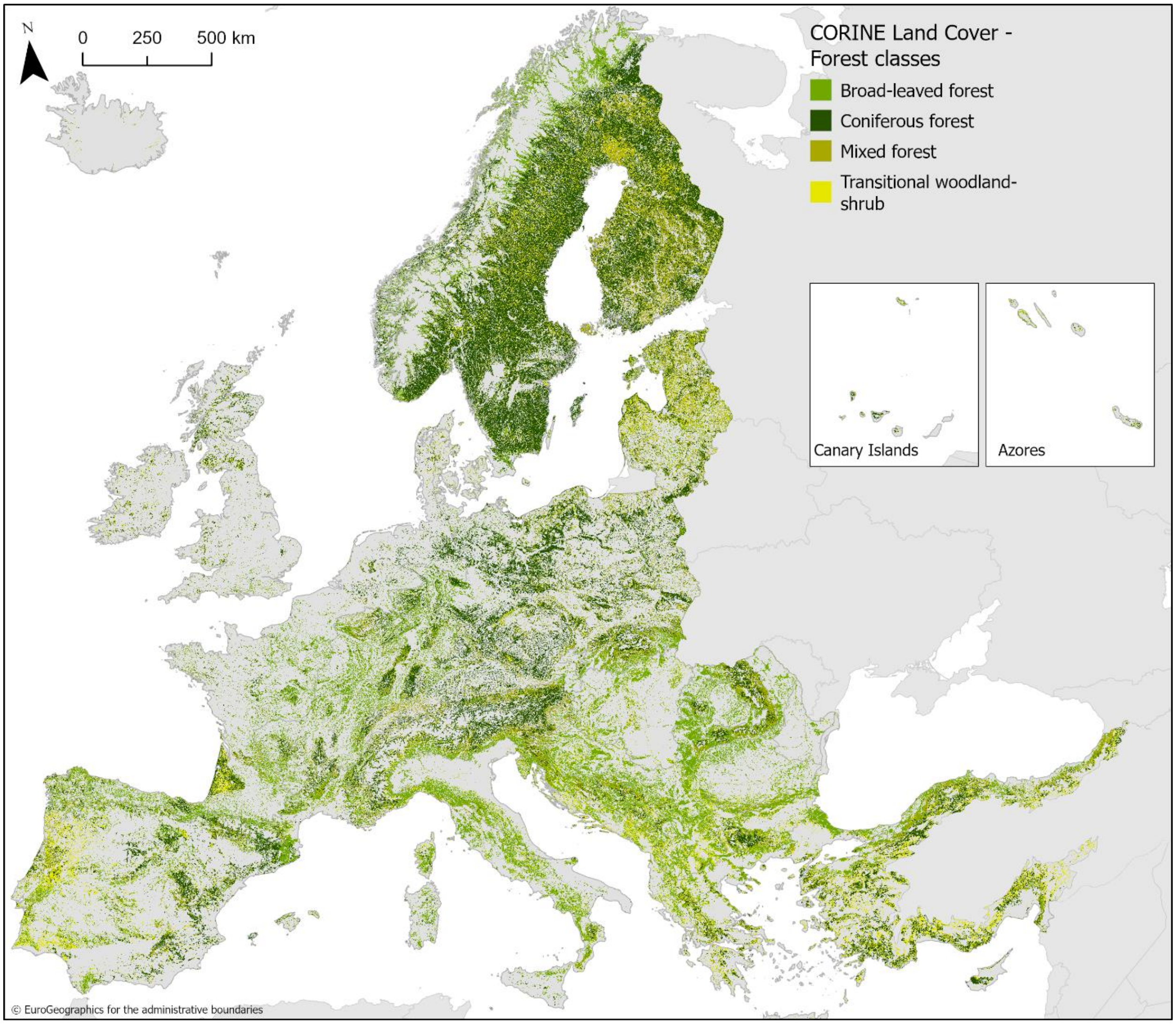

Supplementary Figure 5. Forest cover based on the Corine Land Cover data (2018). This study used the CLC land accounting layers 2000 and 2018 and delineated forests based on the presence of the following CLC classes: broad-leaved forest, coniferous forest, mixed forest, and transitional woodland and shrub. This map provides a forest mask with the boundaries and area for mapping the forest condition variables. CLC is maintained and updated by the European Environment Agency and is a reference for area-based statistics on land cover and land use in Europe. CLC datasets for 1990, 2000, 2006, 2012, and 2018 can be downloaded here: https://land.copernicus.eu/pan-european/corine-land-cover. A detailed description of the forest classes is available here: https://land.copernicus.eu/user-corner/technical-library/corine-land-cover-nomenclature-guidelines/html.

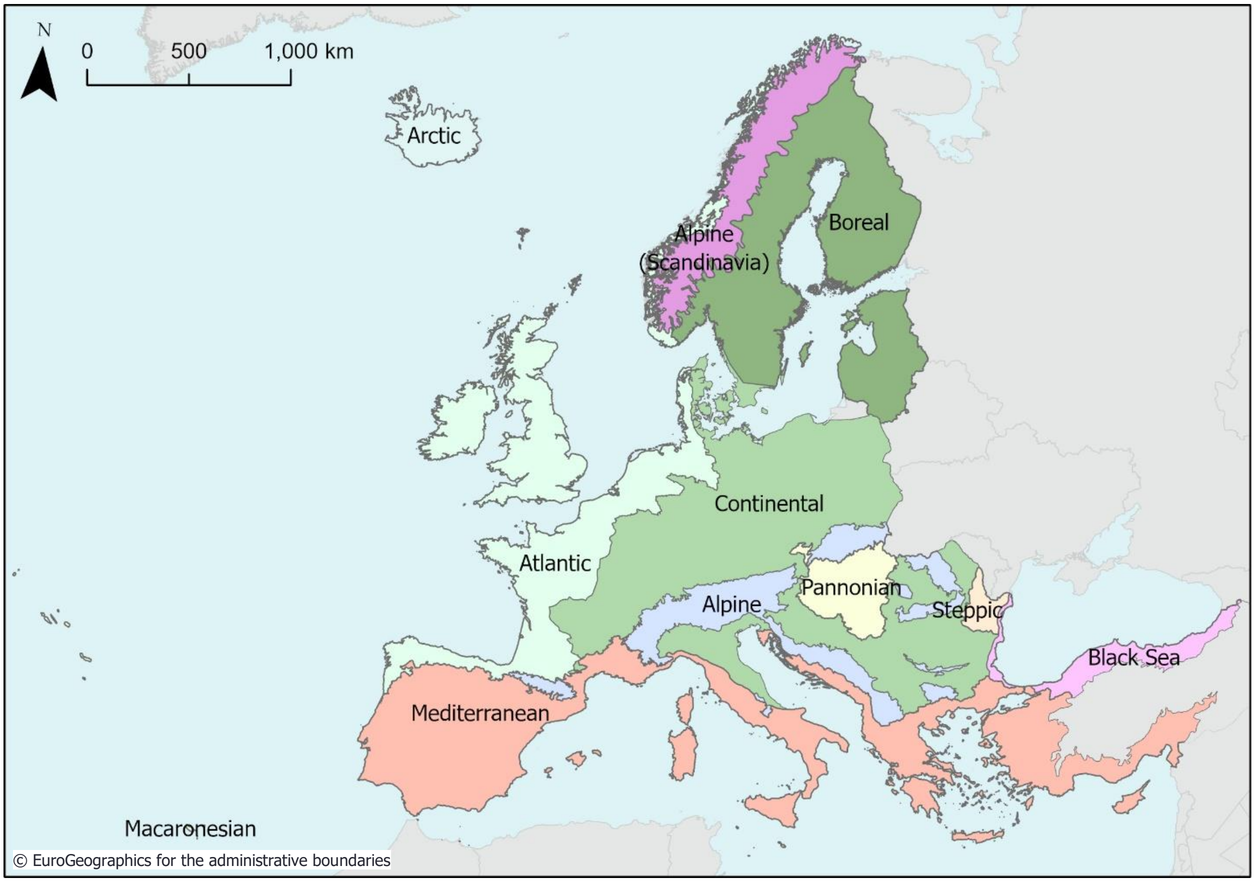

Supplementary Figure 6. Accounting area set by 11 biogeographical regions within the domain covered by Corine Land Cover. The total accounting area is 5,400,442 km^2. The accounting area includes the following countries: Albania, Andorra, Austria, Belgium, Bosnia and Herzegovina, Bulgaria, Croatia, Cyprus, Czechia, Denmark, Estonia, Finland, France, Germany, Greece, Hungary, Iceland, Ireland, Italy, Latvia, Liechtenstein, Lithuania, Luxembourg, Malta, Monaco, Montenegro, Netherlands, North Macedonia, Norway, Poland, Portugal, Romania, San Marino, Serbia, Slovakia, Slovenia, Spain, Sweden, Switzerland, Türkiye, and United Kingdom. The Anatolian biogeographical region of Türkiye is excluded from the analysis. Although it is covered by CLC, and by most of the ecosystem condition variables, we were unable to identify reference sites for this biogeographical region. The five French overseas departments – Martinique, Mayotte, Guadeloupe, French Guiana and Réunion - are not included in this assessment. The biogeographical regions of Europe can be downloaded here: https://www.eea.europa.eu/data-and-maps/data/biogeographical-regions-europe-3

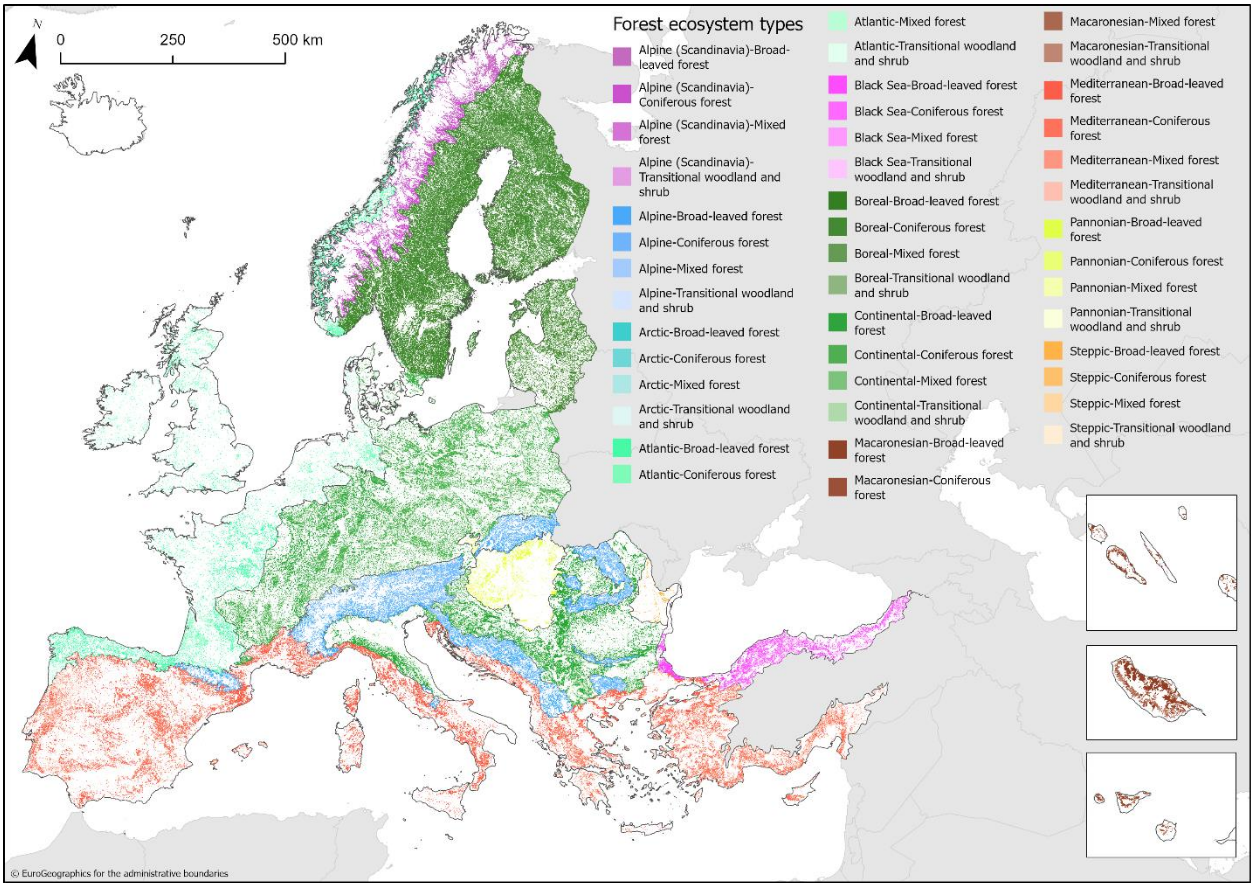

Supplementary Figure 7. Forest ecosystem typology identifying 44 forest ecosystem types which are implemented as the geographic intersection between 11 biogeographic regions and four forest classes, derived from Corine Land Cover. The legend lists all forest types. Naming simply refers to the combination of region and forest class.

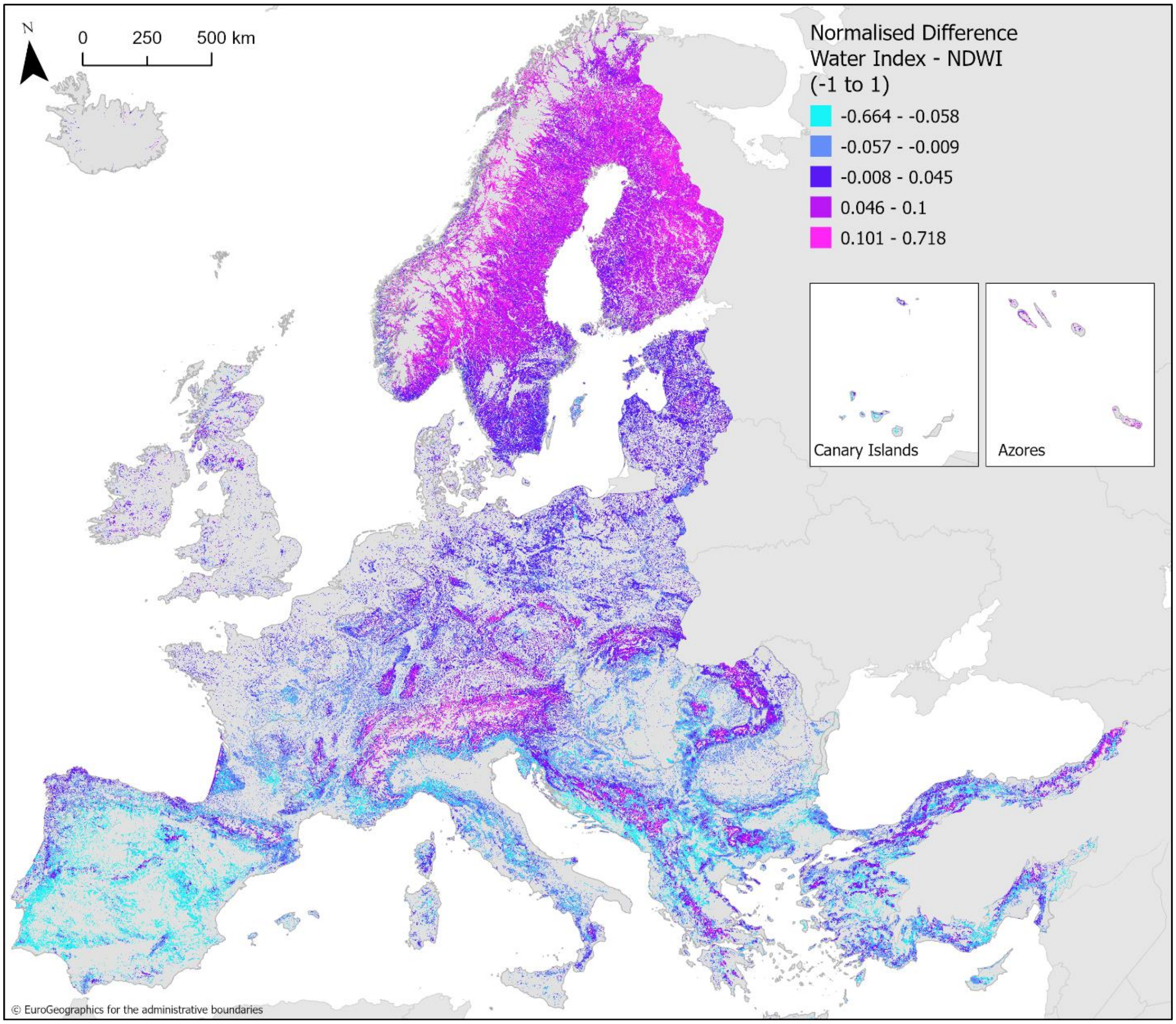

Supplementary Figure 8. Normalized difference water index (NDWI) mapped for forests within the accounting area.

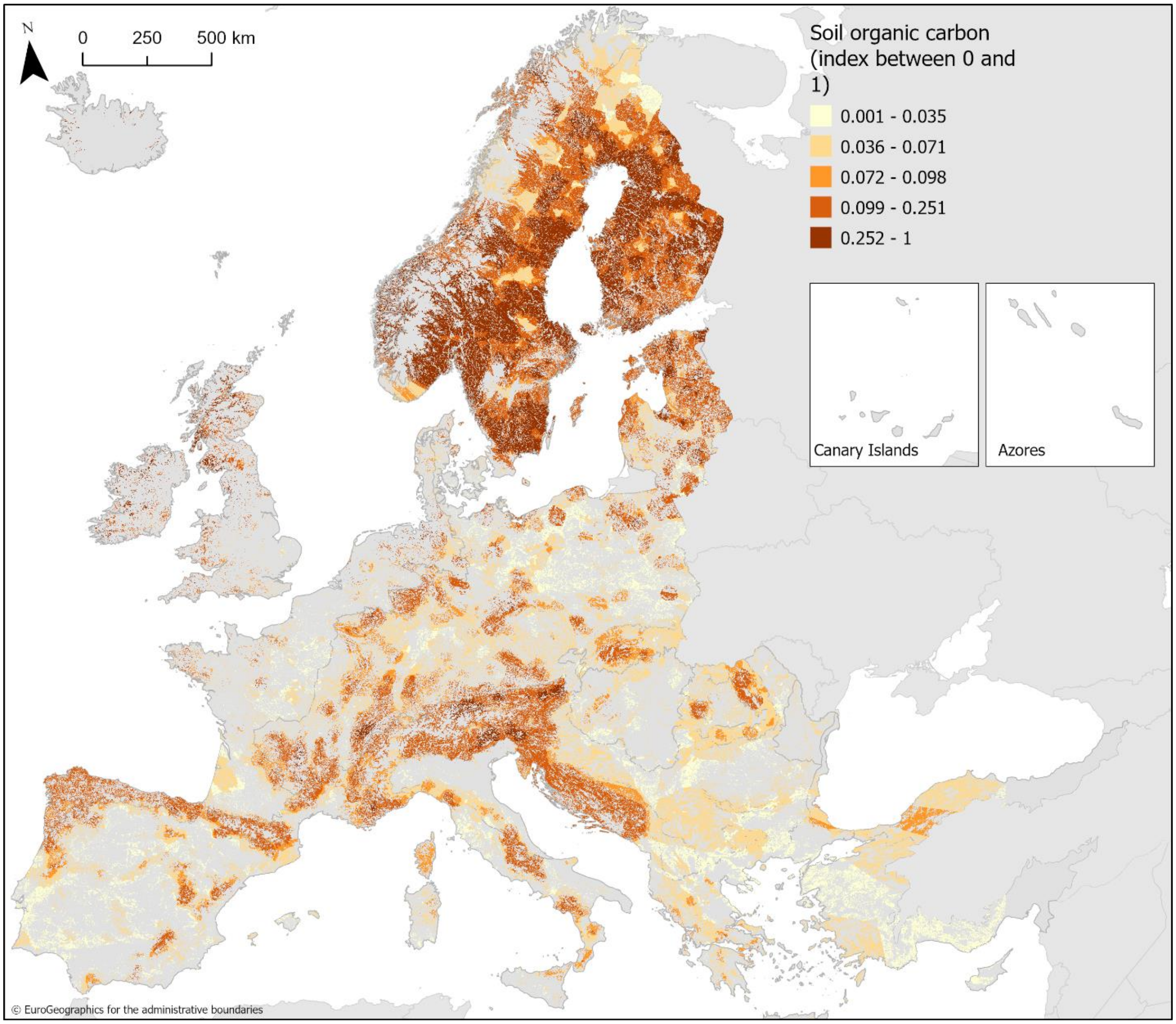

Supplementary Figure 9. Soil organic carbon mapped for forests over the accounting area in 2015. The condition index has been gapfilled for the missing areas in Türkiye and Cyprus using the average value of soil organic carbon per forest ecosystem type. The condition index for the Macaronesian region is based on six indicators and does not include soil organic carbon.

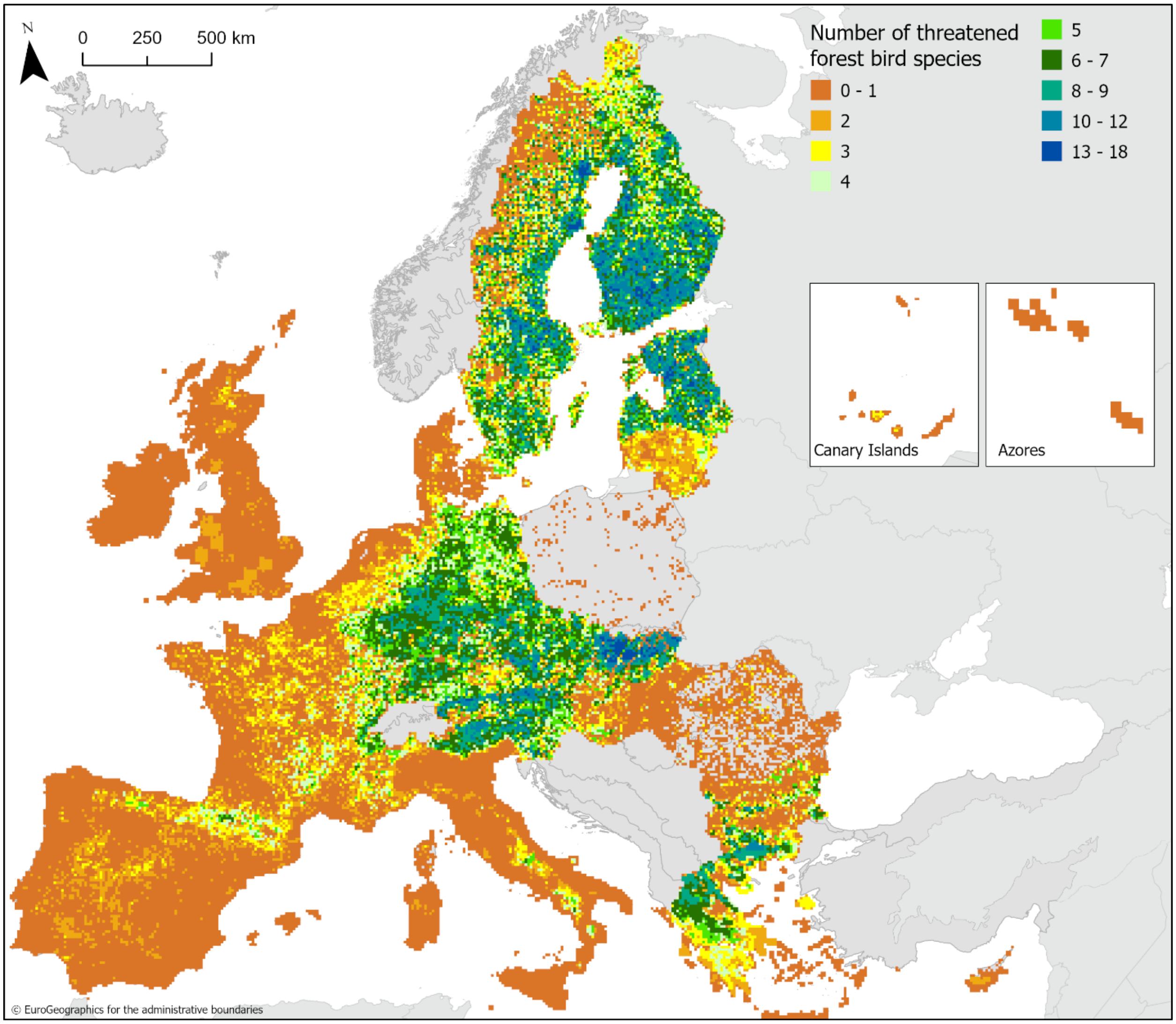

Supplementary Figure 10. Species richness of threatened forest birds reported by EU countries under Article 12 data of the EU Birds Directive for the assessment period 2008 - 2012.

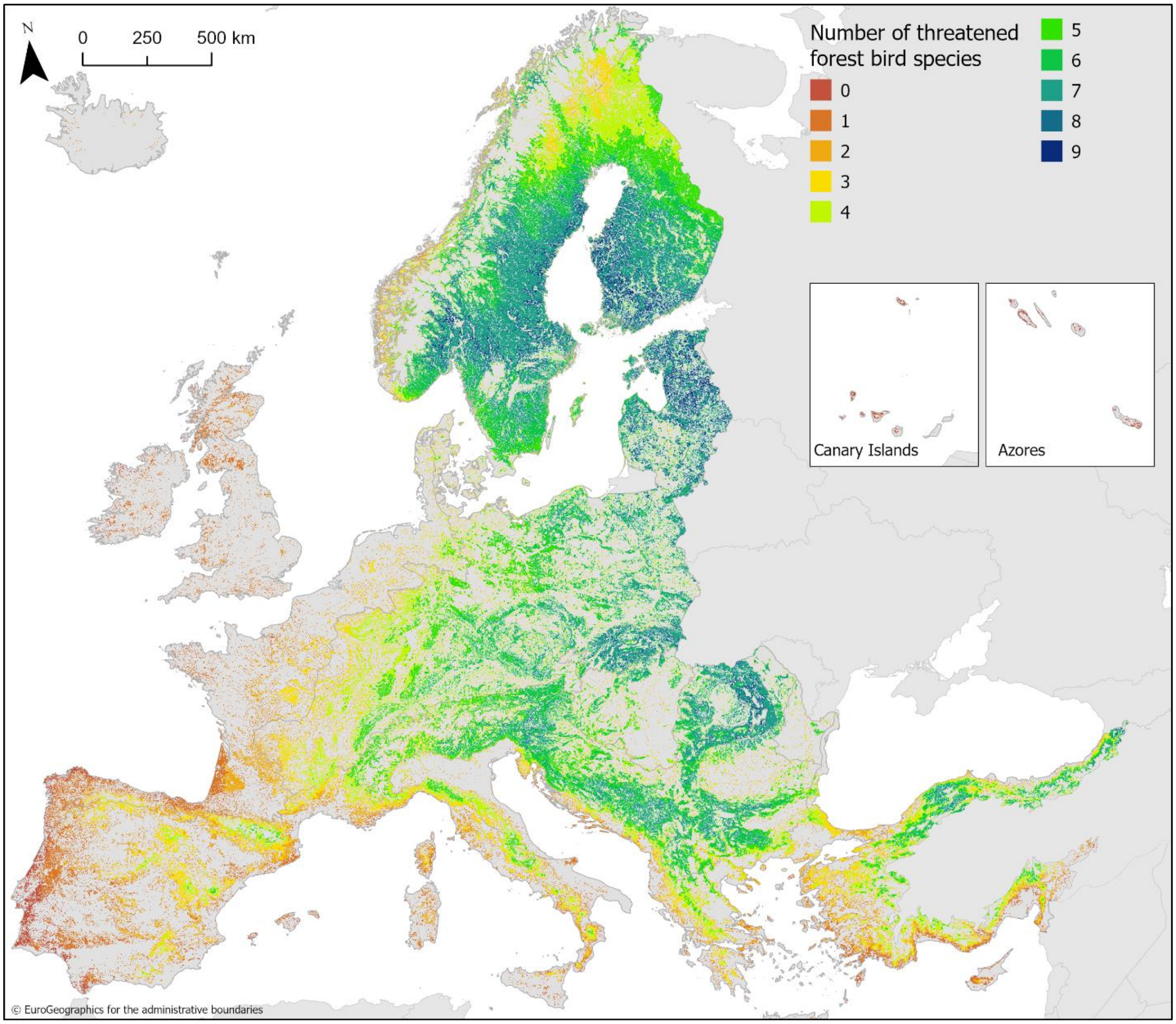

Supplementary Figure 11. Species richness of threatened forest birds in 2018 mapped for the accounting area.

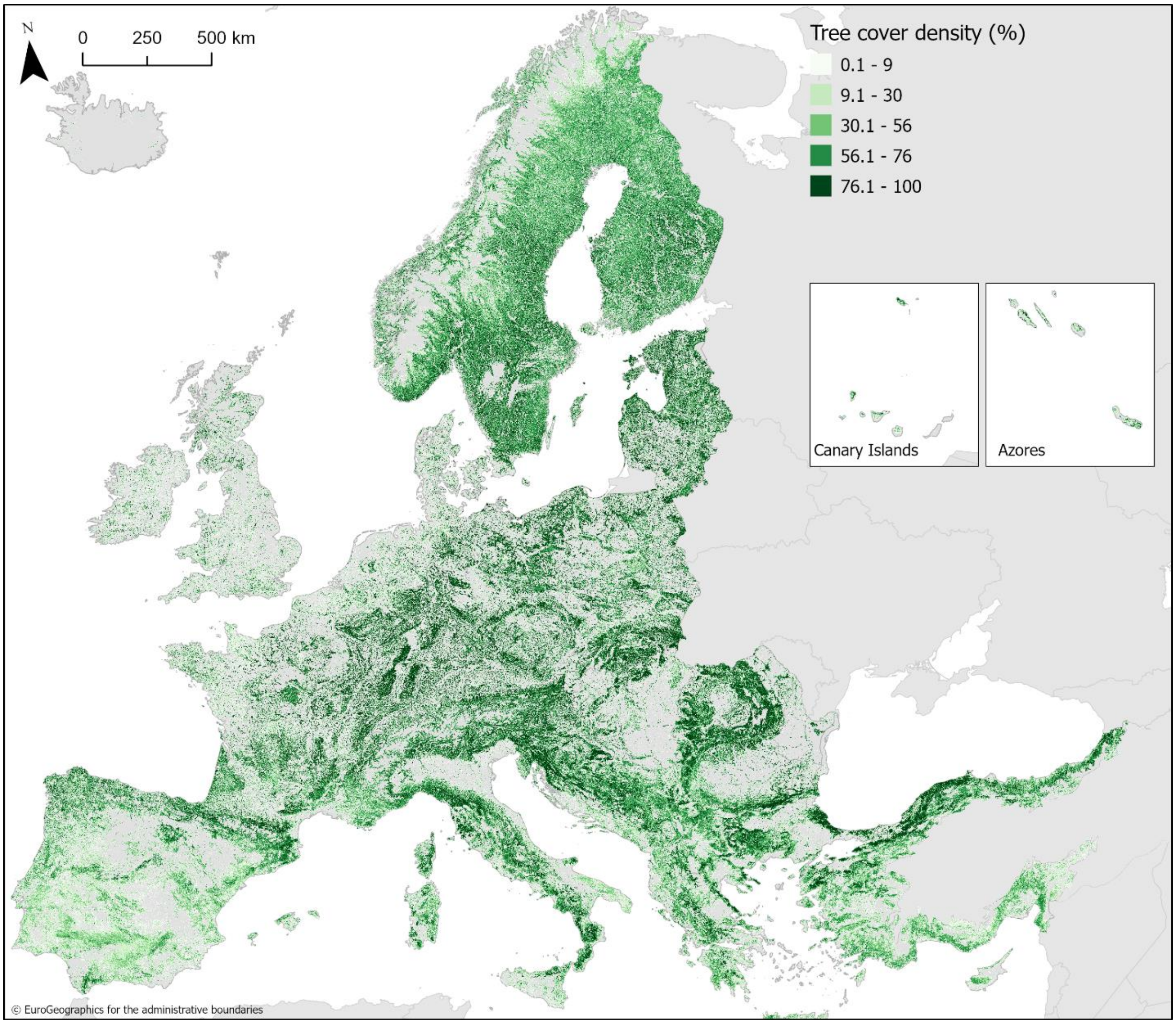

Supplementary Figure 12. Tree cover density for 2018 mapped for forests within the accounting area.

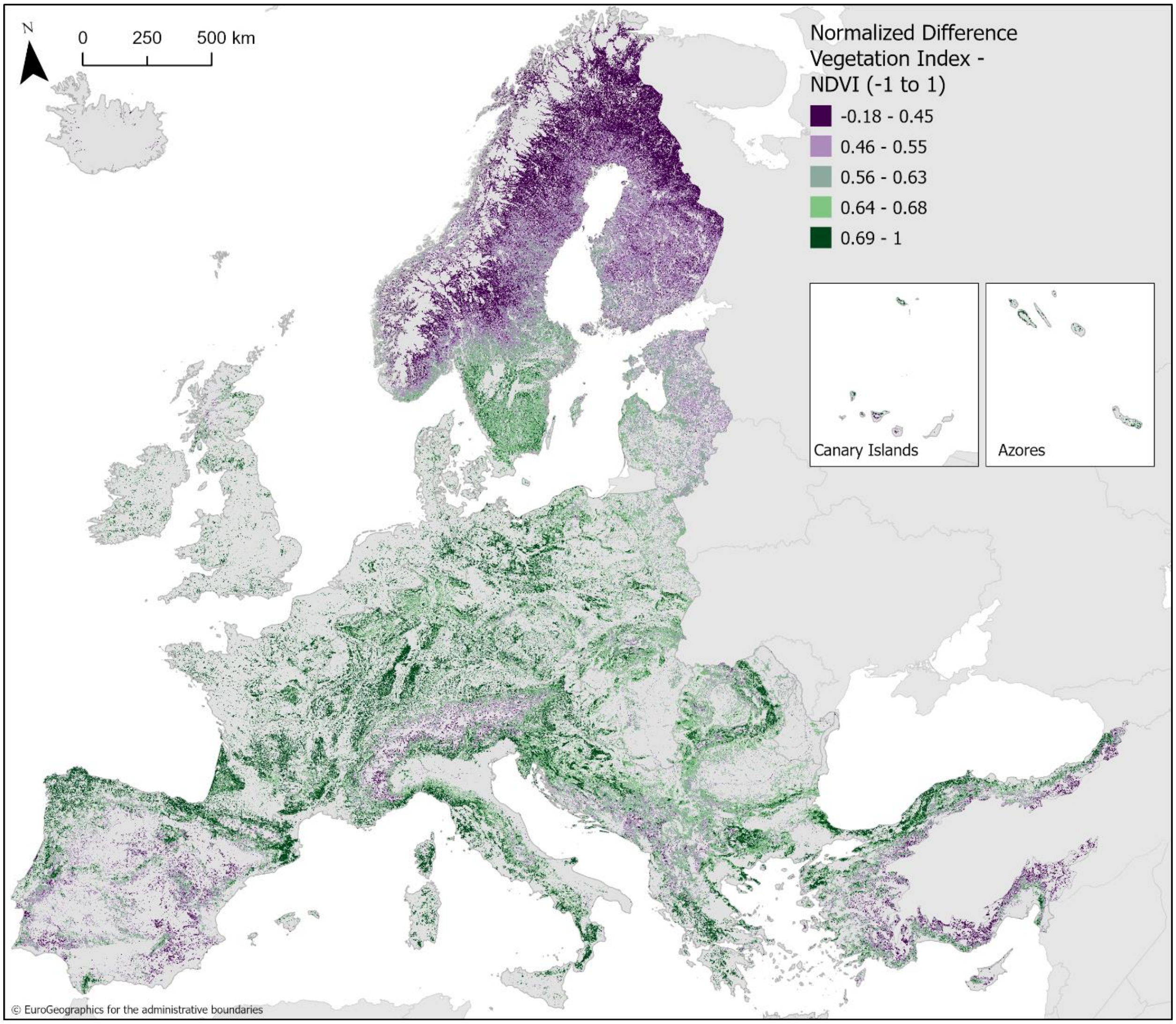

Supplementary Figure 13. NDVI values used for the year 2018 and mapped for forests within the accounting area.

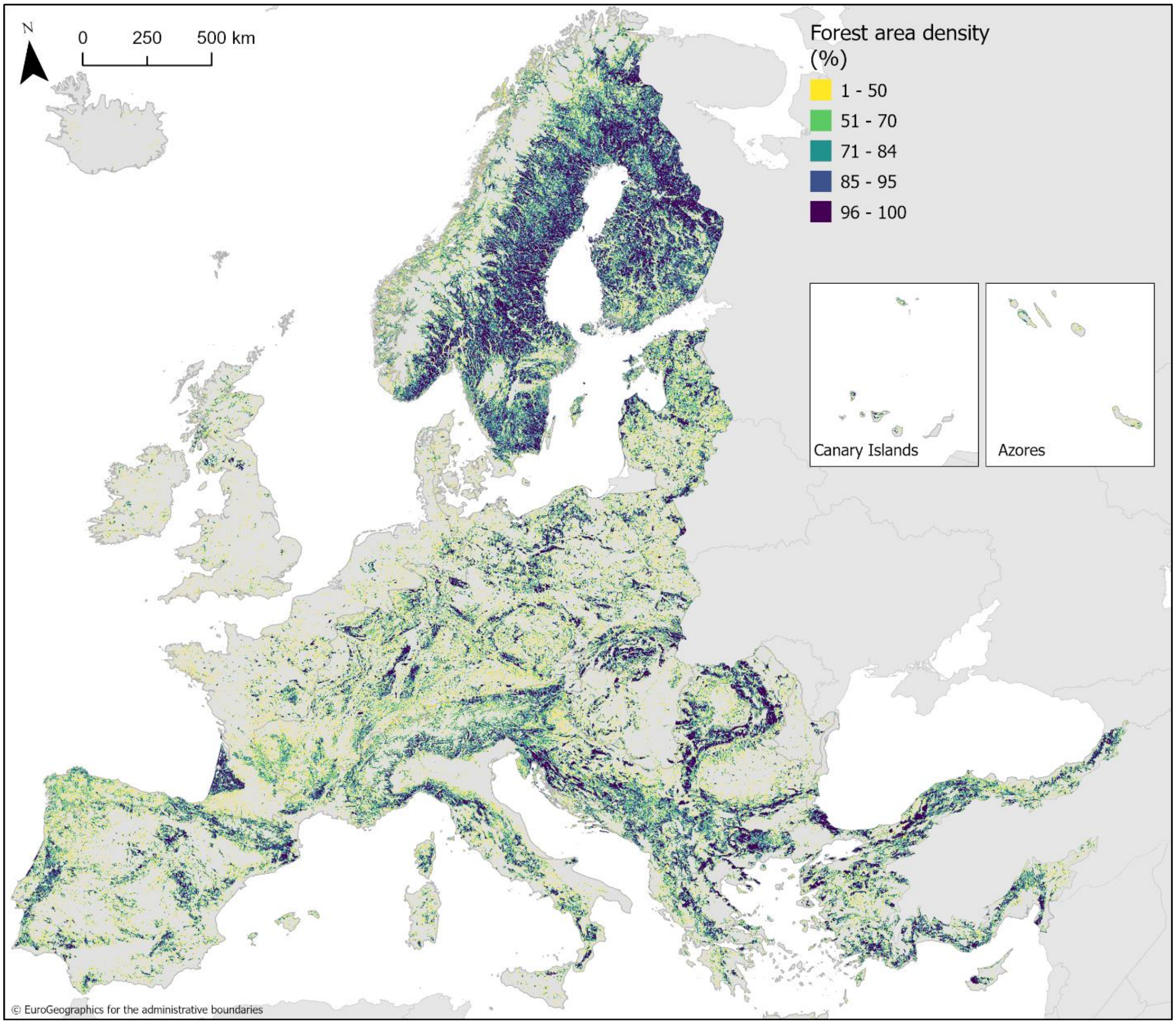

Supplementary Figure 14. Forest connectivity for 2018 mapped for forests within the accounting area.

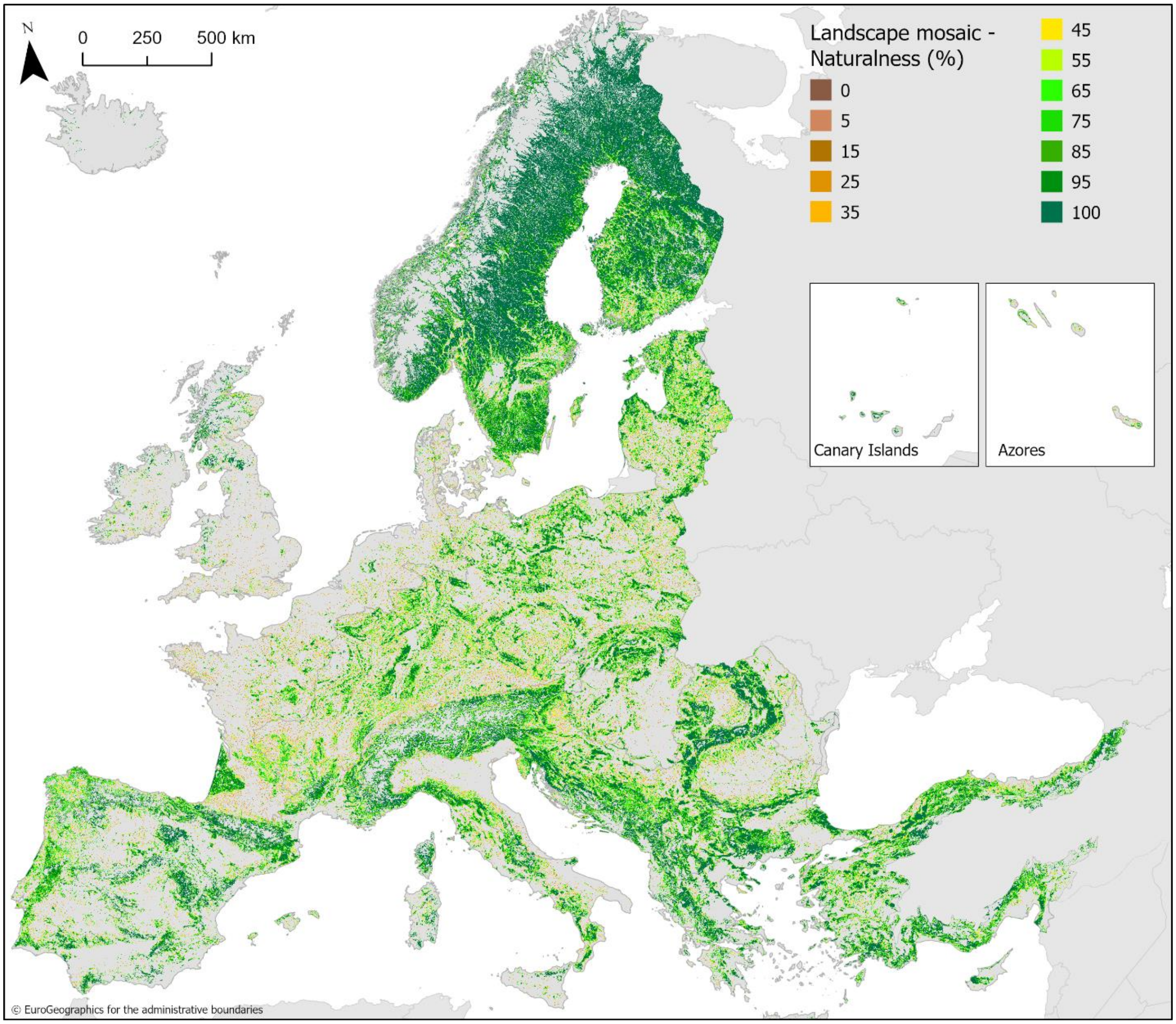

Supplementary Figure 15. Landscape naturalness for 2018 mapped for forests within the accounting area.

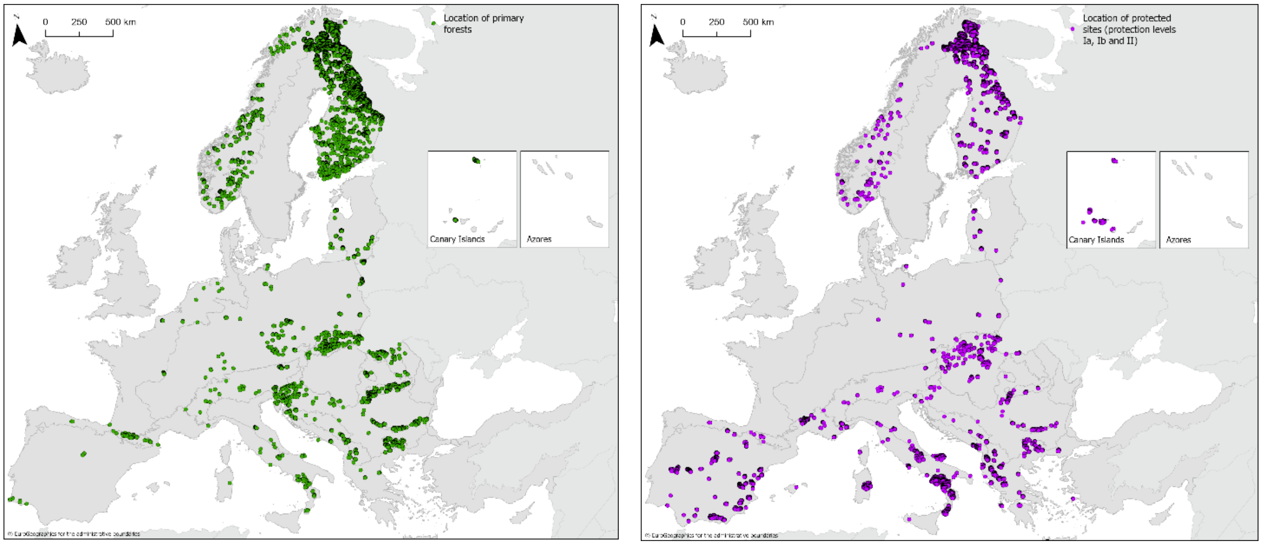

Supplementary Figure 16. Location of reference forests in Europe. Left panel: reference sites covered by primary forest; right panel: reference sites covered by protected sites. Overlap between primary and protected forest occurs frequently but is not quantified here.

Supplementary References

1 Bohn, U., Neuhäusl, R., Gollub, G., Hettwer, C., Neuhäuslová, Z., Raus, Th., Schlüter, H. & Weber, H. (2000/2003): Karte der natürlichen Vegetation Europas / Map of the Natural Vegetation of Europe. Maßstab / Scale 1 : 2 500 000. Münster (Landwirtschaftsverlag)
