# Supplementary material for: Accounting for forest condition in Europe based on an international statistical standard
Source: Nat Commun. 2023 Jun 22;14:3723. doi: 10.1038/s41467-023-39434-0 (PMC10287664; doi:10.1038/s41467-023-39434-0)
Supplement: Supplementary file 3 — Reporting Summary [file 41467_2023_39434_MOESM3_ESM.pdf]

nature portfolio

Corresponding author(s): Joachim Maes

Last updated by author(s): May 22, 2023

Reporting Summary

Nature Portfolio wishes to improve the reproducibility of the work that we publish. This form provides structure for consistency and transparency in reporting. For further information on Nature Portfolio policies, see our Editorial Policies and the Editorial Policy Checklist.

Statistics

For all statistical analyses, confirm that the following items are present in the figure legend, table legend, main text, or Methods section.

n/a	Confirmed	
☐	☒	The exact sample size (*n*) for each experimental group/condition, given as a discrete number and unit of measurement
☒	☐	A statement on whether measurements were taken from distinct samples or whether the same sample was measured repeatedly
☐	☒	The statistical test(s) used AND whether they are one- or two-sided<br>*Only common tests should be described solely by name; describe more complex techniques in the Methods section.*
☐	☒	A description of all covariates tested
☐	☒	A description of any assumptions or corrections, such as tests of normality and adjustment for multiple comparisons
☐	☒	A full description of the statistical parameters including central tendency (e.g. means) or other basic estimates (e.g. regression coefficient) AND variation (e.g. standard deviation) or associated estimates of uncertainty (e.g. confidence intervals)
☐	☒	For null hypothesis testing, the test statistic (e.g. *F*, *t*, *r*) with confidence intervals, effect sizes, degrees of freedom and *P* value noted<br>*Give P values as exact values whenever suitable.*
☒	☐	For Bayesian analysis, information on the choice of priors and Markov chain Monte Carlo settings
☒	☐	For hierarchical and complex designs, identification of the appropriate level for tests and full reporting of outcomes
☐	☒	Estimates of effect sizes (e.g. Cohen's *d*, Pearson's *r*), indicating how they were calculated

Our web collection on statistics for biologists contains articles on many of the points above.

Software and code

Policy information about availability of computer code

Data collection

No software was used to collect data.

Data analysis

The datasets used in this paper are maps with either standard vector formats (ESRI shapefiles) or raster formats (geotiff). All the datasets can be visualized and processed in commonly used GIS software such as ESRI ArcGIS Pro or QGIS.

Soil organic carbon: We used Gaussian kriging, available as tool in ESRI ArcGIS Pro 2.8 to spatially interpolate the LUCAS SOC sampling data.

Forest birds: The number of threatened forest bird species was modelled using the “glm” procedure in the "stats" library of R. Model validation was performed with the R package 'caret' and the procedure 'createDataPartition' to create a random dataset with 70% of the observations for calibration and 30% for validation (Kuhn, M. (2008). Caret package. Journal of Statistical Software, 28(5). The model was made in Rstudio Version 1.4.1717 which uses R version 4.1.3.

Forest area density (FAD): We used the forest typology map and calculate FAD for each grid cell as the proportion of all forest grid cells within a neighbourhood area with a size of 23×23 grid cells or 529 hectares, which is centered over the grid cell. This process is repeated for all grid cells resulting in a new map of the same dimensions but showing forest area density values for the analysed neighbourhood of 529 hectare over each forest grid cell. This processing scheme (FAD 6-class) is available in the open-source software GuidosToolbox version 3.1.

Landscape naturalness: We aggregated the 38 terrestrial Corine Land Cover classes into three main land cover types: agriculture, natural and developed. In analogy to forest connectivity, the degree of naturalness is for each grid cell derived by assessing the proportion of natural grid cells within a neighbourhood area of 529 hectares. Finally, the per-pixel grid cell values of naturalness (in [0, 100] %) are grouped into the following twelve categories of naturalness: [100, 95, 85, 75, 65, 55, 45, 35, 25, 15, 5, 0] %. FLandscape naturalness were analysed using a

custom software package Guidos Toolbox version 3.1, developed by co-author Peter Vogt. The software is freely available: https://forest.jrc.ec.europa.eu/en/activities/lpa/gtb//

Primary and protected forests: The primary and protected forests were mapped using ESRI ArcGIS-Pro 2.8. Calculations on forest disturbance inside the primary and protected forests were done using the ArcGIS-Pro 2.8 geospatial toolbox.

Forest condition index: The following specific software and model code has been used to process the datasets and to calculate a forest condition map. The forest condition variables have been remapped to an annual composite map at 100 m resolution and were then aggregated into a single map using ESRI ArcGIS Pro 2.8.

Statistical tests were performed Rstudio Version 1.4.1717 which uses R version 4.1.3.

For manuscripts utilizing custom algorithms or software that are central to the research but not yet described in published literature, software must be made available to editors and reviewers. We strongly encourage code deposition in a community repository (e.g. GitHub). See the Nature Portfolio guidelines for submitting code & software for further information.

Data

Policy information about availability of data

All manuscripts must include a data availability statement. This statement should provide the following information, where applicable:
- Accession codes, unique identifiers, or web links for publicly available datasets
- A description of any restrictions on data availability
- For clinical datasets or third party data, please ensure that the statement adheres to our policy

Four datasets are made available on Zenodo: the maps of the forest condition 2000 and 2018, the forest typology map, a map representing uncertainty of the condition assessment, and the forest extent and condition accounting tables. The data can be downloaded here: https://doi.org/10.5281/zenodo.7741636

Human research participants

Policy information about studies involving human research participants and Sex and Gender in Research.

Reporting on sex and gender	Not applicable
Population characteristics	Not applicable
Recruitment	Not applicable
Ethics oversight	Not applicable

Note that full information on the approval of the study protocol must also be provided in the manuscript.

Field-specific reporting

Please select the one below that is the best fit for your research. If you are not sure, read the appropriate sections before making your selection.

☐ Life sciences ☐ Behavioural & social sciences ☒ Ecological, evolutionary & environmental sciences

For a reference copy of the document with all sections, see nature.com/documents/nr-reporting-summary-flat.pdf

Ecological, evolutionary & environmental sciences study design

All studies must disclose on these points even when the disclosure is negative.

Study description	The study makes an assessment of the condition of forests in Europe. It integrates publicly available, spatially-explicit data (maps) into a geographical information system (GIS) to define different forest types, to delineate forest reference areas, to map seven ecosystem condition variables, and to aggregate these variables into a single forest condition index.
Research sample	The study area includes 1,964,211 km2 of forest situated on the European continent. The area of forest has been delineated using Corine Land Cover, a reference dataset for area-based statistics on land cover and land use in Europe. The study uses publicly available data that are collected and reported because of legal requirements or that are part of regular monitoring programs, for instance Copernicus, the EU's initiative on earth observation. These datasets have been resampled to a spatial resolution of 100 m and combined into a forest condition index.
Sampling strategy	The sampling strategy for collecting data on the distribution and extent of forests is based on the the usage of available forest reference datasets: corine land cover and the biogeographical regions of Europe. These datasets are made available by the European Environment Agency and are used for official reporting and statistics by EU countries. They are subject to quality control and assurance. The sampling strategy to select and quantify forest condition variables is based on the guidance provided by the System of

Environmental-Economic Accounting - Ecosystem Accounting (SEEA EA), a recent global statistical standard. The selection of forest condition variables is guided by the SEEA ecosystem condition typology, a hierarchical classification consisting of six classes grouped into three main groups: abiotic, biotic and landscape-level ecosystem characteristics. The data underpinning the ecosystem condition variables are described in the Data collection of this report.

Data collection	We used the following data in this study. The forest ecosystem typology is based on Corine Land Cover https://land.copernicus.eu/pan-european/corine-land-cover and the distribution of biogeographical regions of Europe https://www.eea.europa.eu/data-and-maps/data/biogeographical-regions-europe-3. Vegetation water content - Normalized difference water index (NDWI) is available at https://developers.google.com/earth-engine/datasets/catalog/LANDSAT_LC08_C01_T1_8DAY_NDWI. We used the Topsoil Organic Carbon Content for Europe for the year 2003 (OCTOP 2003) resolution by the European Soil Data Centre (ESDAC) of the Joint Research Centre https://esdac.jrc.ec.europa.eu/content/octop-topsoil-organic-carbon-content-europe and the soil organic carbon content based on the Land Use and Coverage Area frame Survey (LUCAS) topsoil data 2015; https://esdac.jrc.ec.europa.eu/content/lucas2015-topsoil-data). The species richness of threatened forest birds was modelled based on the following datasets: bird species distribution maps collected under Article 12 of the EU Birds directive as dependent variable https://sdi.eea.europa.eu/catalogue/srv/eng/catalog.search#/metadata/7c2dd14f-60b6-4009-aca8-5d20300479a9 ; climate data (annual mean temperature, temperature seasonality, annual precipitation, precipitation seasonality) https://www.worldclim.org/data/worldclim21.html; corine land cover data, and NDVI for summer: https://developers.google.com/earth-engine/datasets/catalog/MODIS_MCD43A4_006_NDVI. Data on tree cover density are available from the Copernicus Land Monitoring Service for the years 2012, 2015 and 2018 (Supplementary Figure 12). https://land.copernicus.eu/pan-european/high-resolution-layers/forests/tree-cover-density. The data for forest productivity - Normalized difference vegetation index (NDVI) are available at https://lpdaac.usgs.gov/products/mod13q1v006/. Data on primary forests were sourced from the European Primary Forest Database (EPFD v2.0)21 and UNESCO's Ancient and Primeval Beech Forests of the Carpathians and Other Regions of Europe available at https://www.protectedplanet.net/903141). We used the World Database of Protected Areas (WDPA) (UNEP-WCMC and IUCN, 2020, www.protectedplanet.net) to map protected forests. The map of tree cover loss is available https://earthenginepartners.appspot.com/science-2013-global-forest. The Eu digital elevation model is provided by Eurostat: https://ec.europa.eu/eurostat/web/gisco/geodata/reference-data/elevation/eu-dem. The E-OBS gridded data are available at : https://surfobs.climate.copernicus.eu/dataaccess/access_eobs.php#datafiles. The map of the potential natural vegetation of the European continent71 can be accessed here https://www.synbiosys.alterra.nl/eurovegmap/<br><br>The data underpinning the forest extent and forest condition variables were downloaded between 31/01/2021 and 15/06/2021 by A.G.B with support of the other co-authors and stored on onedrive for further data analysis.<br><br>We generated the following datasets: the forest typology map, the maps of the forest condition 2000 and 2018, the uncertainty map, and the forest extent and condition accounting tables. These datasets have been deposited in Zenodo under accession code https://doi.org/10.5281/zenodo.7741636
Timing and spatial scale	The total accounting area is defined by the intersection of Europe's biogeographical regions with the spatial extent of the Corine Land Cover (CLC) dataset. The total accounting area is 5,400,442 km2. The accounting area includes the following countries: Albania, Andorra, Austria, Belgium, Bosnia and Herzegovina, Bulgaria, Croatia, Cyprus, Czechia, Denmark, Estonia, Finland, France, Germany, Greece, Hungary, Iceland, Ireland, Italy, Latvia, Liechtenstein, Lithuania, Luxembourg, Malta, Monaco, Montenegro, Netherlands, North Macedonia, Norway, Poland, Portugal, Romania, San Marino, Serbia, Slovakia, Slovenia, Spain, Sweden, Switzerland, Türkiye, and United Kingdom. The Anatolian biogeographical region of Türkiye is excluded from the analysis. Although it is covered by CLC, and by most of the ecosystem condition variables, we were unable to identify reference sites for this biogeographical region. The five French overseas departments – Martinique, Mayotte, Guadeloupe, French Guiana and Réunion - are not included in this assessment. The data have different spatial and temporal scales and resolutions. The above mentioned datasets have been aggregated in a GIS (Geographical Information System) environment to an annual composite map at 100 m spatial resolution.
Data exclusions	None
Reproducibility	In principle, the condition assessment reported in this study can be fully reproduced as it is based on publicly available datasets. Moreover, the datasets used in this study are regularly updated, a requirement for ecosystem accounting.
Randomization	A Mann-Whitney U test to compare the forest condition between 2000 and 2018 by forest type is based on a random selection of 1000 sampling points, sampled from a raster layer. Random sampling was carried out within the boundaries of each forest type. ArcPro 2.8 from ESRI was used to obtain a random sample.
Blinding	Not applicable

Did the study involve field work? ☐ Yes ☒ No

Reporting for specific materials, systems and methods

We require information from authors about some types of materials, experimental systems and methods used in many studies. Here, indicate whether each material, system or method listed is relevant to your study. If you are not sure if a list item applies to your research, read the appropriate section before selecting a response.

Materials & experimental systems

n/a	Involved in the study
☒	☐ Antibodies
☒	☐ Eukaryotic cell lines
☒	☐ Palaeontology and archaeology
☒	☐ Animals and other organisms
☒	☐ Clinical data
☒	☐ Dual use research of concern

Methods

n/a	Involved in the study
☒	☐ ChIP-seq
☒	☐ Flow cytometry
☒	☐ MRI-based neuroimaging
